# Epidemiological Trends and Inequalities in Eye Injuries Among Children and Adolescents Aged 0–19 Years: A Comprehensive Analysis of Global, Regional, and National Data From 1990 to 2021

**DOI:** 10.1155/joph/7411787

**Published:** 2026-03-04

**Authors:** Yang Meng, Yuan Liu, Yuan Ma, Ziye Chen, Chin-Ling Tsai, Tao Li

**Affiliations:** ^1^ State Key Laboratory of Ophthalmology, Zhongshan Ophthalmic Center, Sun Yat-Sen University, Guangdong Provincial Key Laboratory of Ophthalmology and Visual Science, Guangdong Provincial Clinical Research Center for Ocular Diseases, Guangzhou, 510060, Guangdong, China, sysu.edu.cn

**Keywords:** burden, children and adolescents, epidemiology, eye injury, GBD 2021

## Abstract

**Background:**

Eye injury is a leading cause of blindness and vision loss worldwide, with children and adolescents being particularly vulnerable. This study aimed to evaluate the global, regional, and national burden of eye injuries among children and adolescents from 1990 to 2021.

**Methods:**

This is a population‐based analysis using data from Global Burden of Disease Study 2021. Eye injury estimates for children and adolescents aged under 20 years, including incidence and years lived with disability (YLDs), were collected and then analyzed by age, sex, and location. The association between sociodemographic index (SDI) and eye injury burden was also investigated.

**Results:**

From 1990 to 2021, the age‐standardized incidence rate (ASIR) and age‐standardized YLD rate (ASYR) for child and adolescent eye injuries decreased by 21.6% and 21.4%, respectively. In 2021, an estimated 11,547,996 children and adolescents worldwide suffered from eye injuries, causing 84,790 YLDs. Boys and adolescents aged 15–19 years had a higher burden. Regionally, South Asia had the highest number of incident cases in 2021, whereas Australasia reported the highest ASIR and ASYR. Nationally, India had the most incident cases, whereas New Zealand had the highest ASIR and ASYR. The SDI‐based analysis revealed that children and adolescents in higher SDI countries had higher ASIR and ASYR than their counterparts in lower SDI countries. Unintentional injuries were the leading cause of child and adolescent eye injuries globally.

**Conclusions:**

Despite a decline in age‐standardized rates of eye injuries globally, eye injury burden remained substantial for children and adolescents. The burden varied by sex, age, location, and SDI, which highlighted the importance of targeted prevention strategies.

## 1. Background

Eye injury is one of the leading causes of blindness and vision loss throughout the world [1]. Apart from the potential sight‐ and eye‐threatening consequences it may cause, eye injury can also have detrimental effects on patients’ mental well‐being and impose a heavy economic burden on the healthcare system [2, 3].

Children and adolescents are particularly prone to eye injury for various reasons, such as their playful nature, limited ability to assess environmental risks, ongoing development of motor coordination, and more fragile facial morphology [4]. In the United States, one‐third of the patients who visit emergency rooms each year for eye injuries are children and adolescents [5]. Compared to adults, the impact of eye injury in child and adolescence can be more severe, as premature disability leads to prolonged mental and physical suffering for individuals and poses a significant economic burden on society [4, 5]. Therefore, systematically understanding of the epidemiological characteristics of eye injuries in children and adolescents is crucial developing effective prevention strategies.

The Global Burden of Disease Study (GBD) is currently the largest scientific effort measuring global health levels and trends, which estimated the burden of 371 diseases and injuries in 204 countries and territories between 1990 and 2021, including eye injury [6]. Based on GBD 2017 and 2019, previous studies have investigated the global burden of eye injuries across all ages and provided useful information [2, 7]. However, less attention has been paid to the special group of children and adolescents. To our knowledge, the long‐term global burden and trends of eye injury among children and adolescents have not yet been reported.

Therefore, this study aimed to evaluate the global, regional, and national burden of eye injuries in children and adolescents from 1990 to 2021, using the most recent data from the GBD 2021. We also investigated the inequalities in the burden related to sociodemographic development level.

## 2. Methods

### 2.1. Overview

This study is a cross‐sectional population‐based analysis based on GBD 2021. The GBD 2021 is a large‐scale global health study conducted by the Institute for Health Metrics and Evaluation of the University of Washington. It offers a detailed assessment of the health conditions for 371 diseases and injuries across 204 countries and territories from 1990 to 2021, offering valuable data to inform global health policies [6]. The detailed methodology and study protocol of the GBD 2021 have been described in previous GBD 2021 publications [6, 8]. In brief, to estimate the burden of eye injuries, a variety of data sources were used as the input data, including data from emergency department records, hospital records, insurance claims, and population‐representative surveys.

The study abided by the Guidelines for Accurate and Transparent Health Estimates Reporting: the GATHER statement and complied with the principles of the Helsinki Declaration [9]. The GBD 2021 has been approved by the Institutional Review Board Committee of the University of Washington (approval No. STUDY00009060). Since this study used publicly available deidentified data, written informed consent is not required.

### 2.2. Study Population

According to the World Health Organization and previous publications, “children and adolescents” were defined as individuals aged 0–19 years and were further divided into 4 subgroups at 5‐year intervals: 0–4 years, 5–9 years, 10–14 years, and 15–19 years [10–12]. In GBD 2021, there are three causes of eye injury: (1) transport injuries, (2) unintentional injuries, and (3) self‐harm and interpersonal violence, each of which includes several subcategories. The list of all these causes (and their corresponding subcategories) along with the relevant International Classification of Diseases (ICD‐10 and ICD‐9) codes can be found in Table S1.

We accessed the GBD Results Tool on the Global Health Data Exchange website (https://ghdx.healthdata.org/) to gather data on eye injuries in children and adolescents, which included estimates of incidence and years lived with disability (YLD), broken down by sex (female, male, and both) and age groups, from 1990 to 2021. The data were collected at 3 levels: global, regional (across 21 GBD regions), and national (204 countries and territories). YLD measures the years a person lives with a disability, with a single YLD representing 1 year of healthy life lost due to disability or poor health. An overview of the regions and countries (territories) included in GBD 2021 is available in Table S2. Both incidence and YLD estimates for eye injuries in children and adolescents are provided as numbers and age‐standardized rates, along with their corresponding 95% uncertainty intervals (UIs).

The GBD 2021 also provides the sociodemographic index (SDI) data for the 204 countries and territories. SDI is commonly used to assess the development status of a region and has a strong correlation with health outcomes. It is a composite measure that combines three key indicators: lag‐distributed income per capita, mean education for individuals aged 15 and older, and the fertility rate for females under 25. The SDI is calculated as the geometric mean of these three indices. It scales between 0 and 1, with 0 representing the lowest theoretical development level, whereas one indicating the highest. In this study, we conducted an SDI‐based analysis to assess SDI‐related inequalities in the burden of eye injuries among children and adolescents.

### 2.3. Trend Analysis

The numbers of incident cases and YLDs, with the corresponding age‐standardized rates, were the primary indicators used to describe the epidemiology of eye injury among children and adolescents. The trends in the disease burden of eye injuries among children and adolescents over the study period were evaluated using the average annual percentage changes (AAPCs), which were calculated through the joinpoint regression method. In the joinpoint regression analysis, the optimal number of joinpoints was determined through the Monte Carlo permutation test, with a maximum of five potential joinpoints. The AAPC is a widely used metric to assess the overall trend of a disease indicator (e.g., incidence rate and YLD rate) over a defined time period, such as 1990–2021 in this study. If both the AAPC value and the lower limit of its 95% confidence interval (CI) are greater than 0, it indicates an overall increasing trend in the disease burden. Conversely, if the AAPC value and the upper limit of its 95% CI are both less than 0, it suggests a decreasing trend. If the 95% CI of AAPC includes 0, it suggests that the disease burden remains stable throughout the analysis period.

### 2.4. Burden Prediction

We further performed a predictive analysis to project the future burden of eye injuries among children and adolescents aged 0–19 years from 2022 to 2040. In this study, the Bayesian age‐period‐cohort model was adopted to forecast such burden, as it is well‐suited for handling complex data commonly encountered in large‐scale epidemiological analyses (e.g., GBD 2021) [13]. As a derivation of the classic age‐period‐cohort model, this method is widely recognized as an important approach to projecting trends in future disease burden [14, 15].

The statistical analyses and visualizations for this study were performed using R software (version 4.3.3) and GraphPad Prism software (version 9.5.1).

## 3. Results

### 3.1. Global Trends

Globally, from 1990 to 2021, the age‐standardized incidence rate (ASIR) and age‐standardized YLD rate (ASYR) of eye injuries among children and adolescents both showed a declining trend. Specifically, the ASIR decreased from 553.68 (95% UI, 393.36–767.33) per 100,000 population in 1990 to 434.24 (95% UI, 305.17–603.22) per 100,000 population in 2021, with an AAPC of −0.79 (95% CI, −0.92 to −0.67). The ASYR also decreased from 4.06 (95% UI, 1.22–8.91) to 3.19 (95% UI, 0.96–6.99) per 100,000 population from 1990 to 2021, with an AAPC of −0.79 (95% CI, −0.92 to −0.67) (Table 1). Accordingly, the global number of child and adolescent eye injury cases was 11,547,996 (95% UI, 8,096,231 to 16,072,199) in 2021, resulting in 84,790 (95% UI, 25,430 to 186,142) YLDs, a 7.8% and 7.7% decrease, respectively, compared to 1990 (Table 1).

**TABLE 1:** Global and regional burden of eye injuries among children and adolescents, 1990–2021.

	Incidence					YLDs				
	1990		2021			1990		2021		
	Cases	AISR per 100,000	Cases	ASIR per 100,000	AAPC, 1990–2021	Number	ASYR per 100,000	Number	ASYR per 100,000	AAPC, 1990–2021
Global	12522046 (8898127–17358806)	553.68 (393.36–767.33)	11547996 (8096231–16072199)	434.24 (305.17–603.22)	−0.79 (−0.92–−0.67)	91900 (27647–201532)	4.06 (1.22–8.91)	84790 (25430–186142)	3.19 (0.96–6.99)	−0.79 (−0.92 to −0.67)

*Sex*										
Female	4214396 (2961793–5893112)	382.53 (268.91–534.67)	3952599 (2715179–5630467)	308.17 (212.39–437.66)	−0.71 (−0.89 to −0.54)	30954 (9208–68770)	2.81 (0.84–6.24)	29049 (8561–64789)	2.26 (0.67–5.04)	−0.72 (−0.9 to −0.53)
Male	8307650 (5932552–11462521)	717.40 (512.15–989.83)	7595396 (5340251–10540238)	553 (389.7–766.08)	−0.83 (−0.95 to −0.71)	60946 (18480–132425)	5.26 (1.6–11.44)	55741 (16715–121603)	4.06 (1.22–8.84)	−0.83 (−0.95 to −0.71)

*Region*										
Andean Latin America	122719 (91060–163848)	648.10 (480.55–866)	121323 (86116–165328)	511.05 (362.96–695.78)	−0.85 (−0.97 to −0.72)	900 (276–1878)	4.76 (1.46–9.92)	891 (268–1917)	3.75 (1.13–8.07)	−0.84 (−0.96 to −0.72)
Australasia	106003 (74190–151069)	1687.81 (1179.35–2405.45)	112656 (78448–158849)	1493.91 (1041.33–2104.14)	−0.40 (−0.43 to −0.37)	779 (216–1606)	12.39 (3.44–25.55)	827 (229–1716)	10.97 (3.04–22.72)	−0.40 (−0.43 to −0.37)
Caribbean	87645 (63414–117590)	579.39 (419.06–777.17)	91534 (66743–122455)	596.26 (435.11–796.75)	0.11 (−0.12–0.34)	644 (192–1359)	4.26 (1.27–8.99)	672 (201–1413)	4.38 (1.31–9.20)	0.11 (−0.11–0.33)
Central Asia	213615 (158141–285681)	685.24 (506.27–918.56)	194854 (141706–262765)	571.36 (415.34–771.35)	−0.53 (−1.06 to 0)	1570 (458–3250)	5.04 (1.47–10.44)	1432 (417–3006)	4.20 (1.22–8.81)	−0.53 (−1.06 to 0)
Central Europe	435044 (298610–626484)	1095.15 (752.83–1574.48)	206084 (138966–301428)	863.89 (583.15–1261.63)	−0.82 (−1 to −0.65)	3197 (890–6707)	8.05 (2.25–16.87)	1515 (415–3254)	6.35 (1.74–13.63)	−0.82 (−0.99 to −0.64)
Central Latin America	592769 (426883–818937)	718.24 (517–992.66)	466807 (325012–654803)	545.9 (380.18–764.47)	−0.80 (−1.09 to −0.52)	4355 (1274–9143)	5.2 (1.54–11.08)	3431 (985–7402)	4.01 (1.15–8.63)	−0.8 (−1.09 to −0.52)
Central Sub‐Saharan Africa	131778 (95568–177087)	425.33 (305.81–576.10)	258001 (183400–353470)	352.81 (250.23–485.14)	−0.42 (−2.39 to 1.59)	966 (303–2044)	3.12 (0.97–6.64)	1893 (572–4099)	2.59 (0.78–5.62)	−0.42 (−2.38 to 1.58)
East Asia	1854002 (1048261–3241264)	389.2 (224.32–674.46)	1234626 (653990–2278545)	353.76 (188.33–652.37)	−0.21 (−0.85 to 0.43)	13592 (3697–32350)	2.85 (0.78–6.77)	9054 (2425–22365)	2.59 (0.70–6.39)	−0.21 (−0.84 to 0.43)
Eastern Europe	620665 (444849–864456)	915.37 (656.48–1274.33)	280198 (202009–380721)	594.67 (429.78–805.86)	−1.40 (−1.56 to −1.23)	4561 (1289–9346)	6.73 (1.9–13.78)	2060 (584–4301)	4.37 (1.24–9.11)	−1.39 (−1.56 to −1.23)
Eastern Sub‐Saharan Africa	620057 (444108–876743)	571.56 (406.29–814.75)	894477 (631968–1222701)	394.21 (278.21–539.6)	−1.35 (−2.28 to −0.41)	4543 (1387–9645)	4.19 (1.28–8.94)	6562 (1993–14084)	2.89 (0.88–6.21)	−1.34 (−2.25 to −0.43)
High‐income Asia Pacific	487415 (355279–647317)	950.43 (696.36–1251.82)	256870 (183615–344526)	822.23 (590.85–1095.81)	−0.47 (−0.51 to −0.42)	3582 (1046–7574)	6.98 (2.05–14.74)	1888 (546–4012)	6.04 (1.76–12.8)	−0.47 (−0.51 to −0.42)
High‐income North America	741025 (515891–1023991)	899.27 (626.15–1241.81)	657858 (426616–969842)	717.16 (466.87–1053.45)	−0.77 (−1.00 to −0.54)	5443 (1601–11680)	6.61 (1.94–14.18)	4830 (1401–10666)	5.27 (1.53–11.58)	−0.77 (−1 to −0.55)
North Africa and Middle East	845990 (632304–1105079)	483.19 (360.6–632.19)	1032172 (752636–1381392)	434.73 (317.24–581.4)	−0.40 (−0.92 to 0.13)	6211 (1888–13248)	3.55 (1.08–7.57)	7576 (2312–16310)	3.19 (0.97–6.87)	−0.40 (−0.91 to 0.12)
Oceania	6494 (4626–8888)	195.72 (139.02–268.67)	12116 (8716–16529)	193.17 (138.61–264.37)	−0.31 (−0.54 to −0.07)	48 (14–105)	1.44 (0.42–3.16)	89 (26–195)	1.42 (0.42–3.12)	−0.30 (−0.53 to −0.07)
South Asia	2129817 (1490417–2959586)	397.33 (277.07–554.72)	2358543 (1546433–3440102)	336.91 (222.36–488.13)	−0.65 (−0.81 to −0.49)	15643 (4718–34267)	2.92 (0.88–6.41)	17325 (5031–38626)	2.48 (0.72–5.50)	−0.60 (−0.79 to −0.41)
Southeast Asia	625440 (463917–832219)	284.29 (210.95–378.12)	515598 (375454–692317)	221.64 (161.67–297.04)	−0.86 (−1.57 to −0.14)	4594 (1371–9744)	2.09 (0.62–4.43)	3790 (1114–8177)	1.63 (0.48–3.51)	−0.85 (−1.55 to −0.15)
Southern Latin America	259994 (189669–353244)	1340.7 (978.3–1821.14)	251124 (183929–337797)	1279.61 (938.96–1718.27)	−0.16 (−0.2 to −0.11)	1909 (550–3970)	9.84 (2.84–20.47)	1844 (530–3811)	9.40 (2.71–19.4)	−0.16 (−0.2 to −0.11)
Southern Sub‐Saharan Africa	116726 (83433–158469)	442. (315.66–601.15)	116686 (80149–162840)	371.47 (255.62–517.42)	−0.57 (−0.69 to −0.45)	856 (256–1826)	3.24 (0.97–6.92)	856 (253–1853)	2.73 (0.81–5.89)	−0.57 (−0.69 to −0.45)
Tropical Latin America	740467 (399974–1390647)	1058.27 (572.34–1993.12)	388397 (238916–655525)	575.87 (354.03–974.13)	−1.97 (−2.13 to −1.81)	5415 (1418–13454)	7.74 (2.03–19.23)	2848 (813–6680)	4.22 (1.21–9.91)	−1.96 (−2.12 to −1.80)
Western Europe	1311336 (889780–1885839)	1305.10 (892.54–1863.57)	1044784 (722941–1465689)	1128.74 (785.43–1574.82)	−0.47 (−0.49 to −0.44)	9625 (2887–21370)	9.58 (2.88–21.21)	7677 (2323–16784)	8.29 (2.51–18.08)	−0.46 (−0.49 to −0.44)
Western Sub‐Saharan Africa	473044 (341137–637119)	439.45 (314.17–597.9)	1053287 (745888–1427370)	394.21 (278.04–537.07)	−0.32 (−0.39 to −0.25)	3470 (1056–7409)	3.22 (0.98–6.93)	7728 (2334–16700)	2.89 (0.87–6.27)	−0.32 (−0.39 to −0.25)

*Note:* Within parentheses were 95% uncertainty intervals for cases, numbers, ASIRs, and ASYRs and 95% confidence intervals for AAPCs, respectively.

Abbreviations: AAPC, average annual percentage change; ASIR, age‐standardized incidence rate; ASYR, age‐standardized YLD rate; YLDs, years lived with disability.

From 1990 to 2021, among children and adolescents, the ASIR and ASYR of eye injuries decreased both for females and males. Notably, the ASIR and ASYR were consistently higher in males compared to females (Figures 1(a) and 1(b) and Table 1). Table 2 provides a detailed, age‐stratified breakdown of the burden of eye injuries among children and adolescents in 1990 and 2021. In terms of age distribution, both incident cases and YLDs decreased in all age groups between 1990 and 2021. The greatest reductions in both incident cases and YLDs were observed among the youngest children (aged 0–4 years), with the incident cases decreasing from 2,837,927 (95% UI, 2,202,128 to 3,619,313) in 1990 to 2,271,053 (1,737,119 to 2,935,947) in 2021 and the number of YLDs declining from 20,812 (95% UI, 6611 to 42,566) to 16,663 (5274–34,763). In contrast, the burden of eye injuries increased with age throughout the study period, with the highest burden observed in adolescents aged 15–19 years. In 2021, this age group accounted for 30.6% of the total number of eye injury cases and YLDs among children and adolescents (Figures 1(c) and 1(d) and Table 2).

FIGURE 1Global trends of eye injuries among children and adolescents, 1990–2021. (a) Trends in ASIR of eye injuries among 0–19 year olds. (b) Trends in ASYR of eye injuries among 0–19 year olds. (c) Trends in prevalent cases of eye injuries among 0–19 year olds. (d) Trends in YLD numbers of eye injuries among 0–19 year olds. ASIR, age‐standardized incidence rate; ASYR, age‐standardized YLD rate; YLD, years lived with disability.

**Figure:**
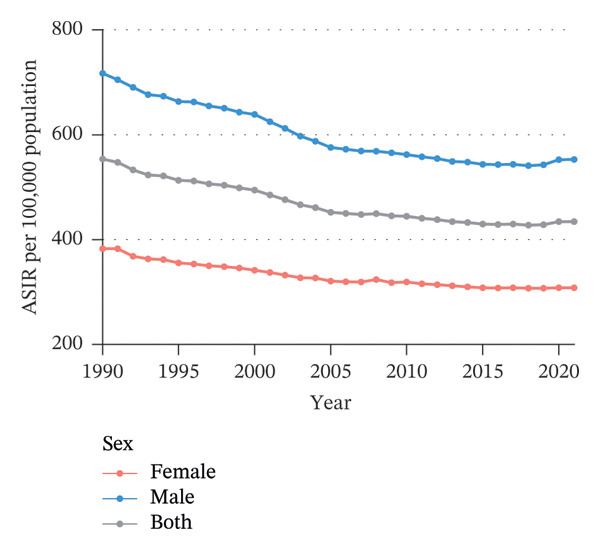
(a)

**Figure:**
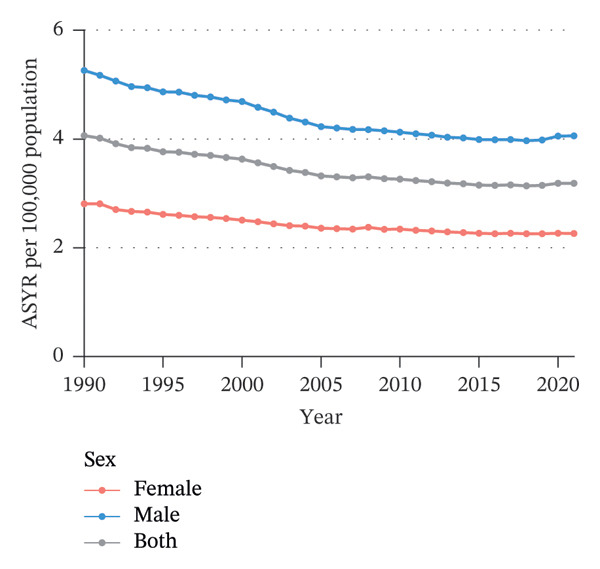
(b)

**Figure:**
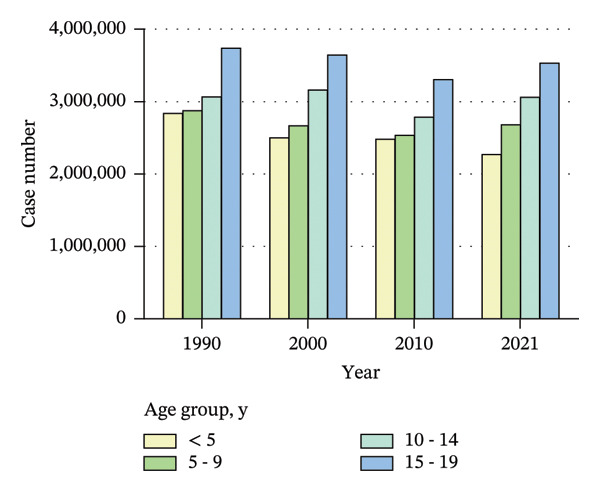
(c)

**Figure:**
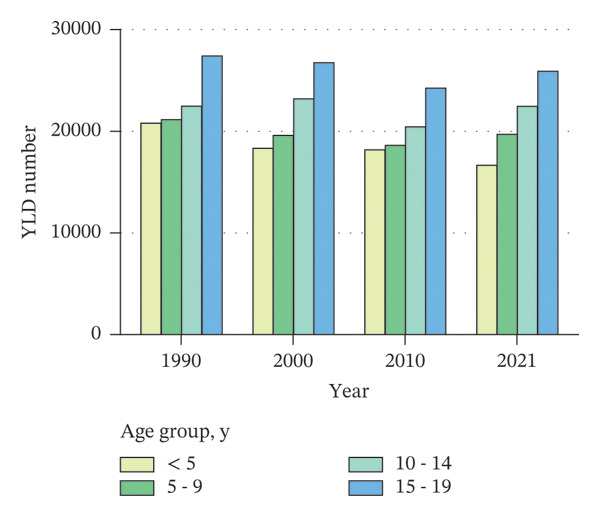
(d)

**TABLE 2:** Age‐stratified burden of eye injuries among children and adolescents, 1990 and 2021.

	Incidence				YLDs			
	1990		2021		1990		2021	
	Cases	Incidence per 100,000	Cases	Incidence per 100,000	Number	YLD rate per 100,000	Number	YLD rate per 100,000
Age group, yrs								
0–4	2837927 (2202128–3619313)	457.78 (355.22–583.82)	2271053 (1737119–2935947)	345.05 (263.93–446.08)	20812 (6611–42566)	3.36 (1.07–6.87)	16663 (5274–34,763)	2.53 (0.80–5.28)
5–9	2878290 (2038761–4065661)	493.25 (349.38–696.73)	2683099 (1873254–3775736)	390.52 (272.65–549.55)	21158 (6316–46675)	3.63 (1.08–8.00)	19732 (5835–42,630)	2.87 (0.85–6.2)
10–14	3067252 (2095423–4193857)	572.59 (391.17–782.90)	3061775 (2088884–4208176)	459.29 (313.35–631.26)	22501 (6600–50787)	4.20 (1.23–9.48)	22467 (6576–50,962)	3.37 (0.99–7.64)
15–19	3,738578 (2561814–5479975)	719.76 (493.20–1055.01)	3532069 (2396974–5152344)	566.05 (384.14–825.72)	27430 (8121–61504)	5.28 (1.56–11.84)	25928 (7745–57786)	4.16 (1.24–926)

*Note:* Within parentheses were 95% uncertainty intervals for cases, numbers, incidence rates, and YLD rates.

Abbreviation: YLDs, years lived with disability.

### 3.2. Regional Trends

Among the 21 regions, South Asia had the highest number of incident cases (2,358,543; 95% UI, 1,546,433 to 3,440,102) and YLDs (17,325; 95% UI, 5031 to 38,626) of eye injuries among children and adolescents in 2021, followed by East Asia. Australasia had the highest ASIR (1493.91/100,000; 95% UI, 1041.33–2104.14) and ASYR (10.97100,000; 95% UI, 3.04–22.72) in 2021, whereas Oceania had the lowest ASIR (193.17/100,000; 95% UI, 138.61–264.37) and ASYR (1.42/100,000; 95% UI, 0.42–3.12) (Table 1). Between 1990 and 2021, the ASIR and ASYR showed significant declines in 16 out of 21 regions. The region with the fastest reduction was tropical Latin America, where the ASIR decreased by 45.6% (from 1058.27/100,000 to 575.87/100,000; AAPC, −1.97) and the ASYR decreased by 45.5% (from 7.74/100,000 to 4.22/100,000; AAPC, −1.96) (Table 1).

In 2021, the ASIR and ASYR for eye injuries in children and adolescents were higher than the global average in 13 regions (e.g., Australasia and Southern Latin America), while 8 regions (e.g., Oceania and Southeast Asia) had lower ASIR and ASYR than the global average (ASIR: 434.24/100,000; ASYR: 3.19/100,000) (Table 1).

### 3.3. National Trends

In 2021, India reported the highest number of incident cases (1,771,988; 95% UI, 1,154,357 to 2,605,157) and YLDs (13,017; 95% UI, 3775 to 29,098) of child and adolescent eye injuries among the 204 countries and territories (Table S3). In the same year, New Zealand had the highest ASIR (1768.97/100,000; 95% UI, 271.03 to 2406.66) and ASYR (12.98/100,000; 95% UI, 3.67–26.39), while Kiribati reported the lowest ASIR (155.69/100,000; 95% UI, 105.07–223.69) and ASYR (1.15/100,000; 95% UI, 0.33–2.53) (Figure 2). Notably, during the study period, the countries with the fastest‐growing ASIR and ASYR for eye injuries among children and adolescents were Afghanistan (AAPC; 1.56 and 1.55), followed by Yemen (1.50 and 1.49) and Libya (1.38 and 1.37). In contrast, Timor‐Leste experienced the fastest declines in ASIR and ASYR (AAPC; −4.70 and −4.68) (Table S3).

FIGURE 2Global map of eye injuries among children and adolescents, 2021, was visualized as heatmaps. Countries (territories) colored red had the highest burden, while those colored blue had the lowest burden. ASIR, age‐standardized incidence rate; ASYR, age‐standardized YLD rate; YLD, years lived with disability.

**Figure:**
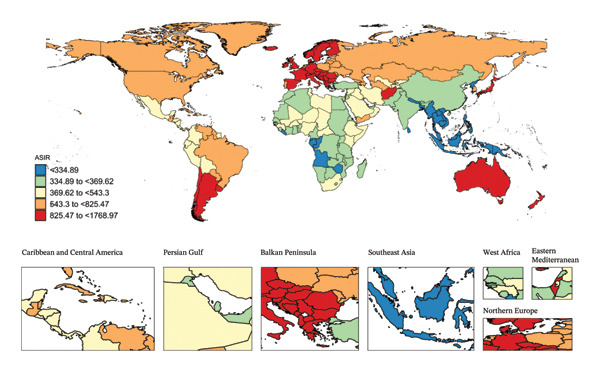
(a)

**Figure:**
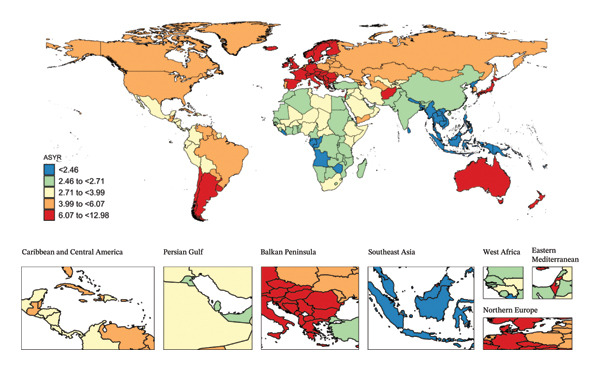
(b)

In 2021, 104 countries (territories) had ASIR and ASYR higher than the global average, such as New Zealand and Italy, while 100 countries (territories) had rates below the global average, including Tonga and Kiribati (Figure 2 and Table S3).

### 3.4. SDI‐Based Trends

In both 1990 and 2021, the ASIR and ASYR were highest in the high SDI group, followed by the high‐middle SDI group, with the remaining three SDI groups showing similar values (Figure 3). Overall, from 1990 to 2021, the ASIR and ASYR for eye injuries among children and adolescents decreased across all five SDI groups. The decreases were statistically significant in all groups, except for the low SDI group, where the decrease approached statistical significance (Table S4). Notably, the ASIR and ASYR in the high SDI group showed an upward trend since 2015.

FIGURE 3SDI‐based trends of children and adolescent eye injuries, 1990–2021. (a) Trends in ASIR of eye injuries in five SDI groups among 0–19 year olds. (b) Trends in ASYR of eye injuries in five SDI groups among 0–19 year olds. SDI, sociodemographic index; ASIR, age‐standardized incidence rate; ASYR, age‐standardized YLD rate; YLD, years lived with disability.

**Figure:**
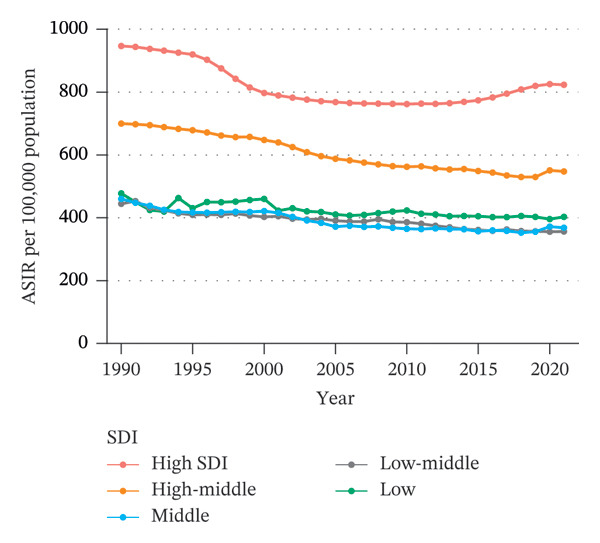
(a)

**Figure:**
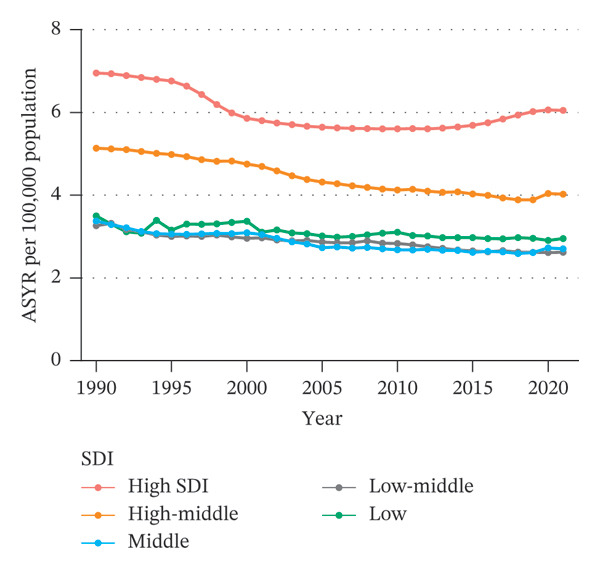
(b)

At the national level, we also analyzed the relationship between each country’s SDI value and eye injury burden in 2021 using Spearman regression. The results of the national‐level analysis are consistent with the group‐based analysis: generally, for low‐, low‐middle‐, and middle‐SDI countries (SDI < 0.71), the ASIR and ASYR remain relatively stable as SDI increases. However, for high‐middle‐ and high‐SDI countries (SDI > 0.71), the ASIR and ASYR increase rapidly with increasing SDI (Figure 4).

FIGURE 4Correlation between children and adolescent eye injury burden and SDI at the national level, 2021. The fitted curves from Spearman regression analysis demonstrate the association between ASIR (a) and ASYR (b) of eye injuries among 0–19 year olds and SDI at the national level in 2021. SDI, sociodemographic index; ASIR, age‐standardized incidence rate; ASYR, age‐standardized YLD rate; YLD, years lived with disability.

**Figure:**
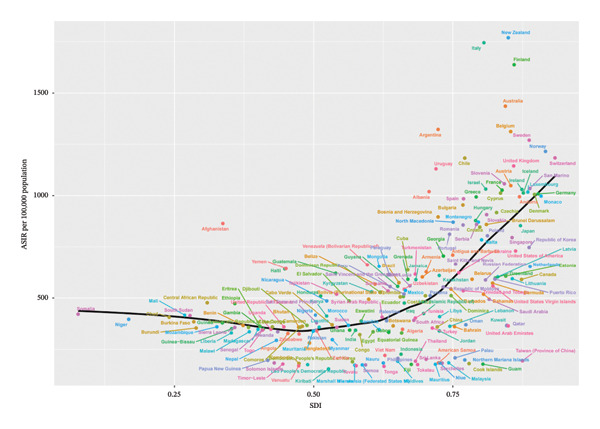
(a)

**Figure:**
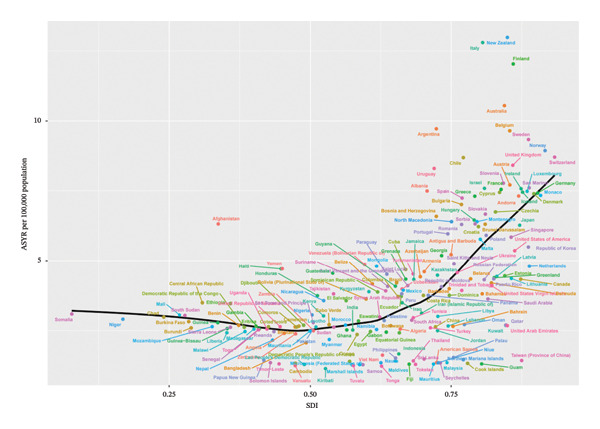
(b)

### 3.5. Main Causes of Eye Injuries

Globally, unintentional injuries (ASIR, 407.26/100,000; ASYR, 2.99/100,000) were the leading cause of eye injuries among children and adolescents in 2021, followed by self‐harm and interpersonal violence (ASIR, 22.14/100,000; ASYR, 0.16/100,000) and transport injuries (ASIR, 4.84/100,000; ASYR, 0.04/100,000) (Figure 5 and Table S5). Regionally, the patterns were largely similar. In 2021, Australasia had the highest ASIR (1468.57/100,000) and ASYR (10.78/100,000) for child and adolescent eye injuries due to unintentional injuries. In contrast, self‐harm and interpersonal violence were the most prominent causes of eye injuries among children and adolescents in North Africa and Middle East (ASIR, 75.20/100,000; ASYR, 0.55/100,000). High‐income North America, on the other hand, reported the highest ASIR (13.93/100,000) and ASYR (0.10/100,000) related to transport injuries (Figure 5 and Table S5).

FIGURE 5The main causes of eye injuries among children and adolescents, 2021. Bar charts demonstrate the composition of ASIR (a) and ASYR (b) of eye injuries among 0–19 year olds globally in 2021, stratified by main causes. ASIR, age‐standardized incidence rate; ASYR, age‐standardized YLD rate; YLD, years lived with disability.

**Figure:**
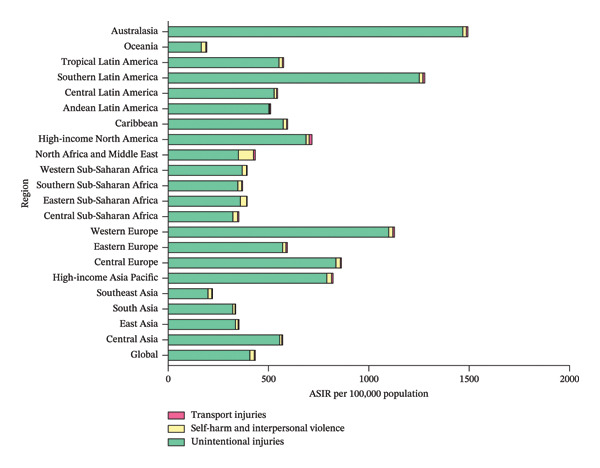
(a)

**Figure:**
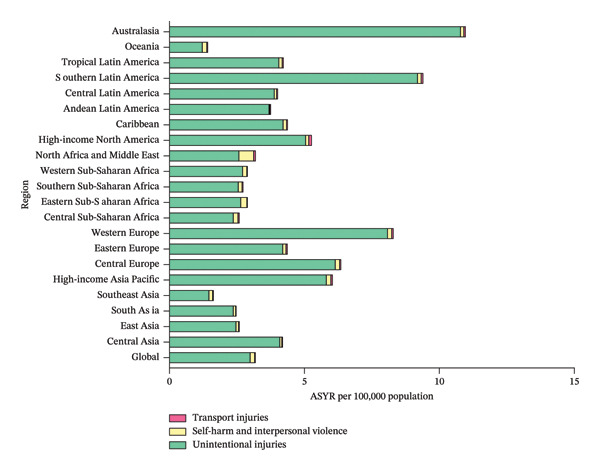
(b)

### 3.6. Burden Prediction

Globally, it is projected that the ASIR and ASYR of eye injuries among children and adolescents will increase between 2022 and 2040 (Figures 6(a) and 6(c)). Specifically, based on past trends, the ASIR and ASYR will increase to 461.04/100,000 and 3.36/100,000 by 2040. Accordingly, the number of expected incident cases and YLDs will also rise: by 2040, an estimated 12.02 million children will experience eye injuries, leading to more than 87,000 YLDs (Figures 6(b) and 6(d)).

FIGURE 6Projected global burden of eye injuries in children and adolescents, 2022–2040, in terms of ASIR (a), incident cases (b), ASYR (c), and YLD numbers (d). ASIR, age‐standardized incidence rate; ASYR, age‐standardized YLD rate; YLD, years lived with disability.

**Figure:**
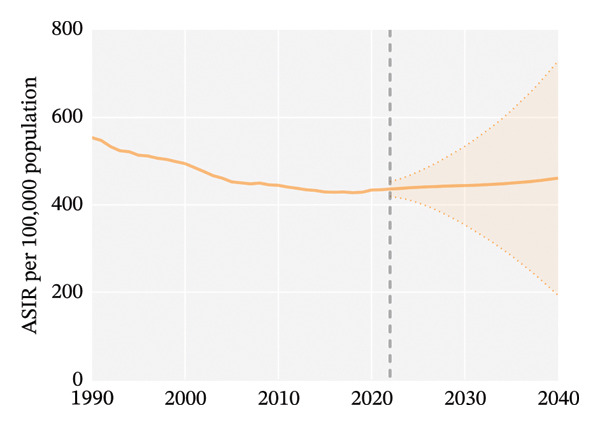
(a)

**Figure:**
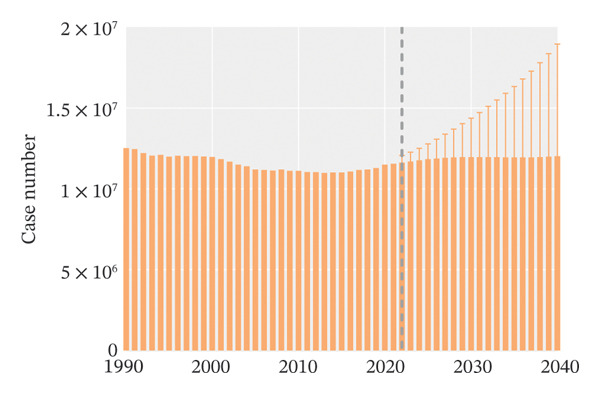
(b)

**Figure:**
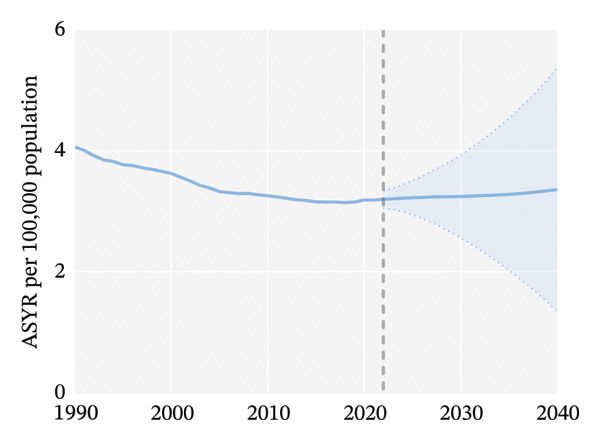
(c)

**Figure:**
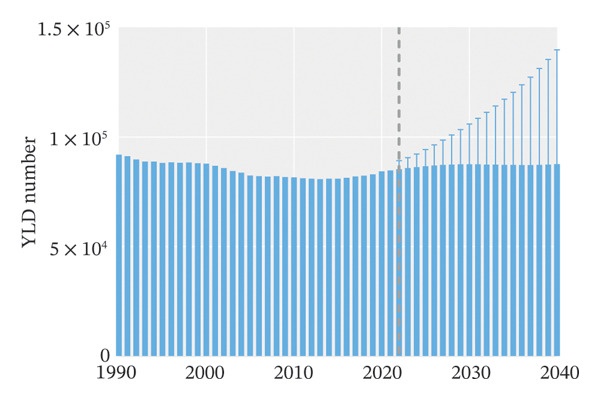
(d)

## 4. Discussion

This study provided a comprehensive and up‐to‐date analysis of the global, regional, and national epidemiology of eye injuries among children and adolescents from 1990 to 2021 using data from GBD 2021. Our findings revealed a general global trend of decreasing burden for eye injuries in this vulnerable population. However, despite this overall improvement, the burden remained substantial, with significant regional and national variations. Furthermore, it is projected that by 2040, the number of children and adolescents affected by eye injuries will continue to rise.

Worldwide, the ASIR and ASYR for eye injuries among children and adolescents showed a significant decline over the study period. Specifically, the ASIR and ASYR decreased by 21.6% and 21.4%, respectively. These declines were likely due to several key factors, including strengthened safety regulations (e.g., toy safety), heightened public awareness of injury prevention among children, parents, and communities, as well as a decline in high‐risk sports activities, among others [16–19]. In contrast, the reductions in the numbers of incident cases (7.8%) and YLDs (7.7%) were much smaller. One key reason for this discrepancy is the global population growth. Over the past few decades, significant progress has been made in improving the health of children and adolescents, and currently there are more children and adolescents in the world than ever before [20, 21]. Even though the ASIR and ASYR declined significantly, the increase in the population size can, to some extent, counterbalance these reductions, leading to smaller declines in incident cases and YLDs. Globally, there were 11,547,996 incident cases and 84,790 YLDs in 2021, suggesting that eye injuries among children and adolescents still pose a major public health threat worldwide.

The present study showed a significant gender disparity in child and adolescent eye injuries, with males consistently exhibiting a higher burden compared to females. This finding was consistent with previous studies reporting a higher incidence of eye injuries among boys [18, 19, 22]. It can be attributed to several facts. For example, boys are typically granted more freedom and tend to spend more unsupervised time outdoors, engaging in more unrestrained and aggressive behaviors compared to girls [22, 23]. The burden of eye injuries also varied across different age groups, with adolescents aged 15–19 years experiencing the highest ASIR and ASYR. This pattern stands in line with the characteristics of this age group, where greater participation in risk‐prone behaviors (e.g., sports, driving, and interpersonal violence) may contribute to a higher incidence of eye injuries [24, 25]. Overall, the findings underscore the importance of targeting public health interventions at males and older adolescents, populations that are at higher risk, to effectively reduce the burden of eye injuries. Interventions could include educational campaigns on eye safety, increased access to protective eyewear, and promoting safer environments in schools and communities to mitigate the risk of injury.

Regionally, the burden of eye injury varied significantly among the 21 regions, with South Asia and East Asia reporting the highest number of injured children and adolescents. This can be attributed to the large population bases in these regions, as well as their rapid transition from predominantly rural societies to highly industrialized ones [2]. Notably, Australasia had the highest ASIR and ASYR in 2021. This finding is consistent with previous studies reporting high incidence rates of eye injuries in Australia and New Zealand, both of which are major countries in Australasia [26–28]. The high incidence of eye injuries in Australia and New Zealand is believed to be primarily associated with environmental and behavioral factors. The environmental factors mainly include high sunlight (ultraviolet) intensity, wildlife‐related injuries, plant‐related injuries, and frequent volcanic activities [2, 29]. Behavioral factors primarily encompass accidental injuries (e.g., sports‐, toy‐, and pet‐related injuries), risky behaviors (e.g., fireworks and bungee jumping), personal assaults, traffic accidents, self‐harm, and more [26–28, 30–32]. At the national level, India stood out with the highest number of incident cases and YLDs, reflecting its large population and possibly insufficient preventive measures for child and adolescent eye injuries. In addition, the notable delay in seeking medical attention is also a concern in India, which reflects, to some extent, a lack of awareness among the population about the severity of eye injuries [33].

The SDI‐based analysis provided further insights into the relationship between economic development and the burden of eye injuries. Our findings indicated that countries with higher SDI values tended to have higher ASIR and ASYR of child and adolescent eye injuries. This observation may be attributed to several factors, including increased vehicle use, industrial accidents, household pets, and engagement in risky activities [5]. Given that most eye injuries in children and adolescents are preventable, further health education, adult supervision, and the adoption of appropriate injury prevention strategies are necessary to reduce the burden of eye injuries in this population, particularly in countries with higher SDI values [34].

The study also investigated the primary causes of eye injuries among children and adolescents globally. Our findings revealed that unintentional injuries were the leading cause, followed by self‐harm and interpersonal violence and transport injuries. This pattern was consistent across most regions, though there were some notable exceptions. For instance, in North Africa and Middle East, the proportion of eye injuries due to self‐harm and interpersonal violence was significantly higher than those in other regions. In GBD 2021, “self‐harm and interpersonal violence”‐related eye injuries include those resulting from self‐harm, interpersonal violence, conflict, and terrorism, as well as police conflict and executions. Evidently, this higher proportion was, at least to some degree, related to the frequent wars and violent political conflicts in the region. Between 1990 and 2021, North African and Middle Eastern countries endured numerous wars and conflicts, including the Gulf War, the Iraq War, the Arab Spring, the Operation Cast Lead (Gaza War I), and the Syrian, Yemen, and Libyan civil wars. These conflicts resulted in a significant number of casualties, including eye injuries [35, 36].

Our projection showed that the global number of children and adolescents affected by eye injuries will increase between 2022 and 2040. This highlights the need for preventive measures and effective interventions to reduce the burden of eye injuries in this vulnerable population. Early intervention strategies, such as promoting eye safety initiatives in schools and homes, are essential for mitigating long‐term outcomes and reducing the socioeconomic burden on families and communities [37].

This study has several implications for the prevention of eye injuries in children and adolescents. First, there is a need for sustained investment in injury prevention programs for children and adolescents, which should focus on education, awareness, and safety measures. Second, while the overall burden of eye injuries in this population has shown some improvement, the variations in eye injury burden across regions and countries highlight the need for targeted interventions to address these disparities. Last but not least, this study underscores the necessity of continuous monitoring and evaluation of eye injuries among children and adolescents. By tracking the burden and causes of eye injuries, policymakers can assess the effectiveness of preventive measures and adjust strategies as needed.

While this study provides a comprehensive analysis of the global burden of eye injuries in children and adolescents, there remain several exciting directions for future research. One promising area is the application of artificial intelligence in predicting prognosis and stratifying the risk of eye injuries. Artificial intelligence models have demonstrated great potentials in other ophthalmic conditions, and similar approaches could be applied to eye injuries in children and adolescents [38, 39]. These models could help identify high‐risk individuals and guide personalized prevention strategies. Additionally, further research is needed to explore the long‐term psychological and economic consequences of eye injuries in this population. Moreover, addressing the factors driving regional disparities in eye injury burden is essential for effective interventions. For example, work on vision impairment and frailty in older adults underscored the role of socioeconomic and health system factors, which could be analogous in pediatric eye injury contexts [40].

Several limitations should be acknowledged for this study. First, the GBD 2021 utilized multiple data sources, and the quality and completeness of the raw data may vary across countries and regions. Second, this study did not investigate the economic costs associated with eye injuries, which would offer additional insights into the broader impact of eye injuries on children and adolescents. Third, this study mainly focused on the overall burden of eye injuries and did not explore their long‐term consequences on the affected individuals. Therefore, future research should aim to address these gaps in order to provide a more holistic understanding of the impact of eye injuries on children and adolescents.

## 5. Conclusions

In conclusion, this study provided a comprehensive analysis of the global, regional, and national burden of eye injuries among children and adolescents from 1990 to 2021. Despite an overall declining trend, the burden remains substantial, with notable variations across regions and countries. Disparities were observed by sex and age, with boys and adolescents aged 15–19 years experiencing a higher burden. Furthermore, children and adolescents in higher SDI countries tended to have a higher risk of eye injuries compared to their counterparts in lower SDI countries. Unintentional injuries were the primary cause of eye injuries globally. Additionally, the global number of children and adolescents affected by eye injuries is projected to further increase by 2040. Policymakers and healthcare providers should prioritize targeted prevention efforts to reduce the burden of eye injuries among children and adolescents, safeguarding their vision and well‐being.

**Nomenclature**
AAPCs: average annual percentage changes
ASIR: age‐standardized incidence rate
ASYR: age‐standardized YLD rate
CI: confidence interval
GBD: Global Burden of Disease Study
ICD: International Classification of Diseases
SDI: sociodemographic index
UIs: uncertainty intervals
YLDs: years lived with disability

## Author Contributions

Yang Meng, Yuan Liu, and Yuan Ma contributed equally as co‐first authors. Yang Meng, Yuan Liu, and Yuan Ma did the study conceptualization, data acquisition, analysis, and visualization and wrote the original manuscript. Yang Meng, Yuan Liu, Yuan Ma, Ziye Chen, Chin‐Ling Tsai, and Tao Li did critical review and edited the manuscript. Tao Li supervised the study.

## Funding

This work was supported by the National Natural Science Foundation of China (grant numbers 82070972 and 82271093), the Key Science & Technology Project of Guangzhou (grant number 202103000045), Guangdong Basic Research Center of Excellence (GBRCE) for Major Blinding Eye Diseases Prevention and Treatment (grant numbers 2024‐RCPY‐009 and 2024‐PIZC‐010), and the Research Funds of the State Key Laboratory of Ophthalmology (grant number 2025OZLHI09).

## Disclosure

All authors reviewed and approved the submitted version of this manuscript. The study sponsor had no role in study design, data analysis, data collection, data interpretation, writing of the report, or decision to submit for publication.

## Ethics Statement

The University of Washington Institutional Review Board Committee approved the GBD 2021 (approval number STUDY00009060). Since this study used publicly available deidentified data, written informed consent is not required.

## Consent

The authors have nothing to report.

## Conflicts of Interest

The authors declare no conflicts of interest.

## Supporting Information

Supporting Table S1: causes of eye injury among children and adolescents in GBD 2021.

Supporting Table S2: a list of the 21 regions and 204 countries (territories) in GBD 2021.

Supporting Table S3: national epidemiology of eye injury among children and adolescents, 1990–2021.

Supporting Table S4: national epidemiology of eye injury among children and adolescents, 1990–2021.

Supporting Table S5: the ASIR and ASYR of eye injury among children and adolescents by region and cause in 2021.

## Supporting information


**Supporting Information** Additional supporting information can be found online in the Supporting Information section.

## Data Availability

The datasets analyzed during the current study are available in the Global Health Data Exchange repository (https://ghdx.healthdata.org/gbd-2021).
