# Supplementary material for: Epidemiological Trends and Inequalities in Eye Injuries Among Children and Adolescents Aged 0–19 Years: A Comprehensive Analysis of Global, Regional, and National Data From 1990 to 2021
Source: J Ophthalmol. 2026 Mar 4;2026:7411787. doi: 10.1155/joph/7411787 (PMC12961164; doi:10.1155/joph/7411787)
Supplement: Supplementary file 1 — Supporting Information Additional supporting information can be found online in the Supporting Information section. [file JOPH-2026-7411787-s001.pdf]

Supplementary materials

Contents

Table S1. Causes of eye injury among children and adolescents in GBD 2021.

Table S2. A list of the 21 regions and 204 countries (territories) in GBD 2021.

Table S3. National epidemiology of eye injury among children and adolescents, 1990-2021.

Table S4. National epidemiology of eye injury among children and adolescents, 1990-2021.

Table S5. The ASIR and ASYR of eye injury among children and adolescents by region and cause in 2021.

Supplementary materials

Table S1. Causes of eye injury among children and adolescents in GBD 2021.

Causes	Subcategories	ICD-10 codes	ICD-9 codes
Transport injuries	Road injuries	V01-V04.9, V06-V80.9, V82-V82.9, V87.2-V87.3	-
	Other transport injuries	V00-V00.8, V05-V05.9, V81-V81.9, V83-V86.9, V88.2-V88.3, V90-V98.8	E800-E807, E830-E838, E840-E849
Unintentional injuries	Falls	W00-W19.9	E880-E886, E888
	Drowning	W65-W70.9, W73-W74.9	E910
	Fire, heat, and hot substances	X00-X06.9, X08-X19.9	E890-E899, E924
	Poisonings	X47-X48.9	E856-E857, E861-E865, E867-E869
	Exposure to mechanical forces	W20-W38.9, W40-W43.9, W45.0-W45.2, W46-W46.2, W49-W52	E916-E922
	Adverse effects of medical treatment	D52.1, D59.0, D59.2, D59.6, D69.5, D70.1-D70.2, D78-D78.8, E03.2, E06.4, E09-E09.9, E16.0, E23.1, E24.2, E27.3, E36-E36.8, E66.1, E88.3, E89-E89.9, G21.0-G21.1, G24.0, G25.1, G25.4, G25.6-G25.7, G72.0, G93.7, G97-G97.9, I95.2-I95.3, I97-I97.9, I98.9, J70.0-J70.5, J95-J95.9, K43-K43.9, K52.0, K62.7, K91-K91.9, K94-K95.8, M87.1, N14-N14.4, N30.4, N65-N65.1, N99-N99.9, P93-P93.8, P96.2, P96.5, R50.2, Y40-Y84.9, Y88-Y88.3	244.0-244.1, 244.3-244.8, 251.3, 253.7, 349.0-349.1, 357.6, 457.0, 518.7, 519.0, 536.4, 539-539.9, 551.2, 552.2, 553.2, 558.1, 564.2-564.4, 569.6, 579.3, 598.2, 779.4-779.5, E870-E876, E878-E879, E930-E949
	Animal contact	W52.0-W62.9, W64-W64.9, X20-X29.9	E905-E906
	Foreign body	W44-W45, W45.3-W45.9, W75-W75.9, W78-W80.9, W83-W84.9	E911-E915
	Environmental heat and cold exposure	L55-L55.9, L56.3, L56.8-L56.9, L58-L58.9, W88-W94.9, W97.9, W99-W99.9, X30-X32.9, X39-X39.9	E900-E902, E926
	Exposure to forces of nature	X33-X38.9	E907-E909
Self-harm and interpersonal violence	Self-harm	X60-X64.9, X66-X83.9, Y87.0	E950-E959
	Interpersonal violence	X85-Y08.9, Y87.1	E960-E969
	Conflict and terrorism	U00-U03, Y36-Y38.9, Y89.1	E979, E990-E999
	Police conflict and executions	Y35-Y35.9, Y89.0	E970-E978

GBD, Global Burden of Disease Study; ICD, International Classification of Diseases.

Table S2. A list of the 21 regions and 204 countries (territories) in GBD 2021.

**GBD regions**	**Countries (territories)**
Andean Latin America	Bolivia (Plurinational State of), Ecuador, Peru
Australasia	Australia, New Zealand
Caribbean	Antigua and Barbuda, Bahamas, Barbados, Belize, Bermuda, Cuba, Dominica, Dominican Republic, Grenada, Guyana, Haiti, Jamaica, Pueto Rico, Saint Kitts and Nevis, Saint Lucia, Saint Vincent and the Grenadines, Suriname, Trinidad and Tobago, United States Virgin Islands
Central Asia	Armenia, Azerbaijan, Georgia, Kazakhstan, Kyrgyzstan, Mongolia, Tajikistan, Turkmenistan, Uzbekistan
Central Europe	Albania, Bosnia and Herzegovina, Bulgaria, Croatia, Czechia, Hungary, Montenegro, North Macedonia, Poland, Romania, Serbia, Slovakia, Slovenia
Central Latin America	Colombia, Costa Rica, El Salvador, Guatemala, Honduras, Mexico, Nicaragua, Panama, Venezuela (Bolivarian Republic of)
Central Sub-Saharan Africa	Angola, Central African Republic, Congo, Democratic Republic of the Congo, Equatorial Guinea, Gabon
East Asia	China, Democratic People's Republic of Korea, Taiwan (Province of China)
Eastern Europe	Belarus, Estonia, Latvia, Lithuania, Republic of Moldova, Russian Federation, Ukraine
Eastern Sub-Saharan Africa	Burundi, Comoros, Djibouti, Eritrea, Ethiopia, Kenya, Madagascar, Malawi, Mozambique, Rwanda, Somalia, South Sudan, Uganda, United Republic of Tanzania, Zambia
High-income Asia Pacific	Brunei Darussalam, Japan, Republic of Korea, Singapore
High-income North America	Canada, Greenland, United States of America
North Africa and Middle East	Afghanistan, Algeria, Bahrain, Egypt, Iran (Islamic Republic of), Iraq, Jordan, Kuwait, Lebanon, Libya, Morocco, Oman, Palestine, Qatar, Saudi Arabia, Sudan, Syrian Arab Republic, Tunisia, Türkiye, United Arab Emirates,Yemen
Oceania	American Samoa, Cook Islands, Fiji, Guam, Kiribati, Marshall Islands, Micronesia (Federated States of), Nauru, Niue, Northern Mariana Islands, Palau, Papua New Guinea, Samoa, Solomon Islands, Tokelau, Tonga, Tuvalu, Vanuatu
South Asia	Bangladesh, Bhutan, India, Nepal, Pakistan
Southeast Asia	Cambodia, Indonesia, Lao People's Democratic Republic, Malaysia, Maldives, Mauritius, Myanmar, Philippines, Seychelles, Sri Lanka, Thailand, Timor-Leste, Viet Nam
Southern Latin America	Argentina, Chile, Uruguay
Southern Sub-Saharan Africa	Botswana, Eswatini, Lesotho, Namibia, South Africa, Zimbabwe
Tropical Latin America	Brazil, Paraguay
Western Europe	Andorra, Austria, Belgium, Cyprus, Denmark, Finland, France, Germany, Greece, Iceland, Ireland, Israel, Italy, Luxembourg, Malta, Monaco, Netherlands, Norway, Portugal, San Marino, Spain, Sweden, Switzerland ,United Kingdom
Western Sub-Saharan Africa	Benin, Burkina Faso, Cabo Verde, Cameroon, Chad, Côte d'Ivoire, Gambia, Ghana, Guinea, Guinea-Bissau, Liberia, Mali, Mauritania, Niger, Nigeria, Sao Tome and Principe, Senegal, Sierra Leone, Togo

GBD, Global Burden of Disease Study.

Table S3. National epidemiology of eye injury among children and adolescents, 1990-2021.

Location	Incidence					YLDs				
	**Number, 1990**	**ASIR per 100000, 1990**	**Number, 2021**	**ASIR per 100000, 2021**	**AAPC, 1990-2021**	**Number, 1990**	**ASYR per 100000, 1990**	**Number, 2021**	**ASYR per 100000, 2021**	**AAPC, 1990-2021**
Afghanistan	28328 (20918 to 37838)	510.17 (375.66 to 683.52)	149194 (89704 to 265682)	863.91 (517.05 to 1544.07)	1.56 (-0.17 to 3.32)	208 (64 to 443)	3.74 (1.14 to 7.99)	1091 (303 to 2529)	6.32 (1.75 to 14.64)	1.55 (-0.17 to 3.3)
Albania	20354 (13893 to 29738)	1410.82 (962.57 to 2062.59)	6433 (4414 to 9189)	1019.41 (699.94 to 1457.17)	-0.97 (-1.27 to -0.67)	149 (42 to 315)	10.36 (2.91 to 21.81)	47 (13 to 99)	7.49 (2.11 to 15.68)	-0.97 (-1.26 to -0.67)
Algeria	57693 (42396 to 76819)	429.74 (315.69 to 572.99)	56323 (40335 to 77212)	347.91 (248.85 to 478.54)	-0.67 (-1.09 to -0.25)	424 (127 to 895)	3.16 (0.95 to 6.67)	414 (122 to 902)	2.56 (0.76 to 5.58)	-0.67 (-1.09 to -0.25)
American Samoa	51 (37 to 70)	217.9 (154.76 to 298)	37 (26 to 52)	184.92 (129.42 to 259.03)	-0.48 (-0.86 to -0.09)	0 (0 to 1)	1.6 (0.47 to 3.46)	0 (0 to 1)	1.36 (0.4 to 2.96)	-0.48 (-0.86 to -0.1)
Andorra	141 (98 to 197)	1030.26 (722.42 to 1421.28)	146 (101 to 204)	994.33 (692.09 to 1376.15)	-0.11 (-0.14 to -0.08)	1 (0 to 2)	7.57 (2.3 to 16.53)	1 (0 to 2)	7.31 (2.21 to 16)	-0.11 (-0.14 to -0.08)
Angola	31062 (22039 to 44524)	549.69 (385.2 to 800.26)	60571 (42350 to 83325)	327.82 (227.98 to 454.65)	-1.2 (-3.02 to 0.65)	228 (70 to 488)	4.03 (1.24 to 8.72)	444 (132 to 958)	2.41 (0.71 to 5.21)	-1.2 (-3.02 to 0.65)

Antigua and Barbuda	157 (113 to 215)	653.71 (470.37 to 891.37)	169 (121 to 231)	710.24 (511.11 to 969.23)	0.18 (0.01 to 0.34)	1 (0 to 2)	4.8 (1.41 to 9.97)	1 (0 to 3)	5.22 (1.52 to 10.81)	0.18 (0.02 to 0.34)
Argentina	192667 (140199 to 262545)	1477.93 (1076.07 to 2013.33)	183344 (132753 to 247760)	1323.7 (960.96 to 1785.23)	-0.36 (-0.42 to -0.3)	1415 (407 to 2947)	10.85 (3.13 to 22.6)	1347 (387 to 2820)	9.72 (2.81 to 20.32)	-0.36 (-0.42 to -0.3)
Armenia	11916 (8813 to 16093)	906.9 (670.28 to 1225.85)	4840 (3483 to 6557)	627.26 (451.95 to 848.76)	-0.66 (-1.16 to -0.15)	88 (26 to 179)	6.66 (1.96 to 13.65)	36 (10 to 74)	4.61 (1.33 to 9.57)	-0.66 (-1.16 to -0.16)
Australia	84615 (58977 to 121581)	1636.6 (1138.83 to 2350.86)	89694 (61841 to 128008)	1436.61 (991.58 to 2047.45)	-0.42 (-0.46 to -0.38)	622 (173 to 1280)	12.02 (3.34 to 24.72)	659 (182 to 1374)	10.55 (2.92 to 21.97)	-0.42 (-0.46 to -0.38)
Austria	22569 (16178 to 30847)	1189.48 (855.5 to 1615.9)	18517 (12987 to 25637)	1048.33 (738.06 to 1444.47)	-0.41 (-0.44 to -0.37)	166 (50 to 355)	8.74 (2.61 to 18.74)	136 (41 to 296)	7.71 (2.33 to 16.72)	-0.4 (-0.42 to -0.38)
Azerbaijan	20762 (15279 to 28109)	666.72 (490.22 to 904.01)	18556 (13337 to 25046)	602.47 (433.81 to 812.08)	-0.42 (-0.77 to -0.07)	153 (45 to 316)	4.9 (1.44 to 10.15)	136 (40 to 284)	4.43 (1.3 to 9.19)	-0.42 (-0.76 to -0.07)
Bahamas	571 (401 to 787)	528.28 (372.5 to 725.73)	609 (427 to 847)	518.83 (365.7 to 716.22)	-0.13 (-0.45 to 0.19)	4 (1 to 9)	3.88 (1.16 to 8.29)	4 (1 to 10)	3.81 (1.13 to 8.25)	-0.13 (-0.45 to 0.19)
Bahrain	758 (541 to 1023)	384.47 (273.68 to 521.69)	1489 (1049 to 2053)	360.9 (254.8 to 495.68)	-0.26 (-0.34 to -0.19)	6 (2 to 12)	2.83 (0.84 to 6.12)	11 (3 to 24)	2.65 (0.78 to 5.8)	-0.26 (-0.33 to -0.19)

Bangladesh	180548 (125871 to 251066)	302.44 (209.31 to 424.48)	174383 (118192 to 249538)	281.58 (191.7 to 400.72)	-0.47 (-0.79 to -0.15)	1326 (394 to 2858)	2.22 (0.66 to 4.82)	1281 (374 to 2827)	2.07 (0.6 to 4.55)	-0.46 (-0.78 to -0.15)
Barbados	469 (330 to 648)	549.67 (388.29 to 756.06)	365 (256 to 505)	543.52 (382.22 to 749.32)	-0.02 (-0.06 to 0.02)	3 (1 to 7)	4.04 (1.19 to 8.73)	3 (1 to 6)	4 (1.17 to 8.62)	-0.02 (-0.06 to 0.02)
Belarus	22785 (16371 to 31700)	722.6 (519.53 to 1004.78)	12113 (8443 to 17019)	591.25 (413.52 to 827.19)	-0.65 (-0.72 to -0.59)	167 (48 to 346)	5.31 (1.51 to 10.96)	89 (25 to 187)	4.35 (1.24 to 9.09)	-0.65 (-0.71 to -0.59)
Belgium	34554 (23419 to 48562)	1377.27 (939.53 to 1925.43)	33803 (22634 to 48104)	1313.27 (885.07 to 1861.1)	-0.17 (-0.3 to -0.03)	254 (76 to 557)	10.12 (3.03 to 22.2)	248 (74 to 553)	9.65 (2.88 to 21.42)	-0.16 (-0.3 to -0.03)
Belize	633 (458 to 856)	619.43 (448.05 to 837.99)	986 (710 to 1343)	567.62 (408.41 to 771.41)	-0.24 (-0.34 to -0.15)	5 (1 to 10)	4.55 (1.37 to 9.58)	7 (2 to 15)	4.17 (1.25 to 8.78)	-0.24 (-0.34 to -0.15)
Benin	11809 (8521 to 15749)	406.87 (290.11 to 550.84)	27531 (19559 to 36970)	366.64 (258.83 to 496.3)	-0.33 (-0.35 to -0.31)	87 (27 to 183)	2.98 (0.91 to 6.36)	202 (61 to 434)	2.69 (0.82 to 5.8)	-0.33 (-0.35 to -0.31)
Bermuda	89 (63 to 123)	561.03 (392.72 to 771.85)	66 (46 to 91)	572.15 (400.32 to 786.58)	0.06 (0.03 to 0.08)	1 (0 to 1)	4.12 (1.22 to 8.84)	0 (0 to 1)	4.21 (1.23 to 9.02)	0.06 (0.03 to 0.08)
Bhutan	1234 (863 to 1714)	360.96 (252.35 to 501.65)	923 (648 to 1283)	357.95 (252.5 to 495.36)	-0.14 (-0.27 to -0.01)	9 (3 to 20)	2.65 (0.77 to 5.71)	7 (2 to 15)	2.63 (0.79 to 5.67)	-0.14 (-0.27 to -0.02)

Bolivia (Plurinational State of)	19045 (13968 to 25326)	569.34 (416.51 to 759.31)	22512 (15993 to 30538)	493.85 (350.97 to 669.4)	-0.47 (-0.5 to -0.43)	140 (42 to 295)	4.18 (1.26 to 8.82)	165 (50 to 355)	3.63 (1.09 to 7.79)	-0.46 (-0.5 to -0.43)
Bosnia and Herzegovina	20686 (14179 to 30347)	1373.51 (942.99 to 2013.15)	6055 (4043 to 8900)	895.97 (598.5 to 1316.9)	-1.53 (-3.1 to 0.08)	152 (42 to 319)	10.08 (2.82 to 21.15)	45 (12 to 96)	6.59 (1.79 to 14.24)	-1.52 (-3.08 to 0.07)
Botswana	2794 (2001 to 3796)	379.08 (270.91 to 516.39)	3356 (2381 to 4577)	364.4 (259.03 to 495.8)	-0.12 (-0.16 to -0.09)	20 (6 to 44)	2.78 (0.83 to 5.94)	25 (7 to 53)	2.67 (0.8 to 5.7)	-0.12 (-0.16 to -0.09)
Brazil	727018 (389775 to 1373755)	1069.91 (574.36 to 2027.99)	371776 (226745 to 632744)	574.31 (350.09 to 979.82)	-2.02 (-2.18 to -1.85)	5316 (1387 to 13243)	7.82 (2.04 to 19.49)	2726 (772 to 6421)	4.21 (1.19 to 9.92)	-2.01 (-2.17 to -1.84)
Brunei Darussalam	964 (709 to 1272)	839.48 (615.97 to 1110.11)	1103 (806 to 1473)	859.13 (630.19 to 1142.91)	0.07 (0.05 to 0.1)	7 (2 to 15)	6.17 (1.83 to 12.86)	8 (2 to 17)	6.31 (1.86 to 13.12)	0.07 (0.04 to 0.09)
Bulgaria	26417 (18451 to 37716)	1099.74 (769.27 to 1568.92)	12501 (8501 to 18161)	954.11 (650.54 to 1383.62)	-0.45 (-0.49 to -0.42)	194 (55 to 398)	8.08 (2.29 to 16.59)	92 (26 to 196)	7.01 (1.95 to 14.93)	-0.45 (-0.49 to -0.42)
Burkina Faso	21948 (15891 to 29456)	384.42 (274.93 to 523.26)	49033 (35370 to 65498)	384.49 (274.99 to 517.67)	0.1 (-0.08 to 0.28)	161 (49 to 342)	2.82 (0.85 to 6.04)	360 (111 to 772)	2.82 (0.87 to 6.08)	0.1 (-0.08 to 0.28)
Burundi	12306 (8897 to 16471)	392.24 (281.32 to 530.72)	25552 (18189 to 34948)	353.98 (251.09 to 486.61)	0.27 (-4.04 to 4.78)	90 (27 to 192)	2.88 (0.86 to 6.17)	187 (57 to 400)	2.6 (0.79 to 5.56)	0.27 (-4.02 to 4.75)

Cabo Verde	818 (588 to 1098)	418.55 (299.44 to 564.85)	716 (504 to 981)	369.57 (261.37 to 503.27)	-0.4 (-0.43 to -0.38)	6 (2 to 13)	3.07 (0.93 to 6.53)	5 (2 to 11)	2.71 (0.81 to 5.79)	-0.4 (-0.43 to -0.37)
Cambodia	14587 (10778 to 19765)	265.68 (195.48 to 362.04)	12119 (8852 to 16210)	181.9 (132.9 to 243.26)	-1.17 (-2 to -0.33)	107 (32 to 225)	1.95 (0.59 to 4.1)	89 (25 to 192)	1.34 (0.38 to 2.87)	-1.16 (-1.99 to -0.33)
Cameroon	22058 (15696 to 30030)	371.23 (261.81 to 511.01)	60962 (43300 to 82754)	362.44 (256.81 to 493.75)	0.05 (-0.01 to 0.1)	162 (49 to 348)	2.72 (0.82 to 5.89)	447 (137 to 968)	2.66 (0.81 to 5.76)	0.05 (-0.01 to 0.1)
Canada	51461 (36794 to 69340)	663.86 (475.07 to 892.95)	49127 (34244 to 67185)	590.35 (412.9 to 804.78)	-0.38 (-0.39 to -0.36)	378 (112 to 807)	4.88 (1.45 to 10.41)	361 (105 to 772)	4.34 (1.27 to 9.27)	-0.37 (-0.39 to -0.36)
Central African Republic	5853 (4246 to 7900)	389.99 (280.32 to 531.22)	13703 (9659 to 19158)	476.85 (335.08 to 668.88)	0.73 (0.26 to 1.21)	43 (13 to 91)	2.86 (0.87 to 6.11)	100 (31 to 217)	3.5 (1.07 to 7.57)	0.73 (0.26 to 1.21)
Chad	15983 (11630 to 21460)	456.74 (327.54 to 620.37)	44136 (32323 to 58481)	406.72 (295.33 to 543.9)	-0.36 (-0.61 to -0.1)	117 (36 to 246)	3.35 (1.04 to 7.1)	324 (100 to 690)	2.98 (0.92 to 6.39)	-0.36 (-0.61 to -0.1)
Chile	52148 (37628 to 71758)	989.05 (712.81 to 1361.24)	57352 (42044 to 78380)	1182.55 (866.98 to 1614.67)	0.58 (0.47 to 0.68)	383 (108 to 797)	7.27 (2.05 to 15.12)	421 (119 to 856)	8.68 (2.45 to 17.66)	0.57 (0.47 to 0.68)
China	1821118 (1025377 to 3201956)	395.1 (226.8 to 688.42)	1213476 (639934 to 2249851)	359.22 (190.31 to 665.57)	-0.21 (-0.86 to 0.45)	13350 (3623 to 31847)	2.9 (0.79 to 6.88)	8898 (2374 to 22041)	2.63 (0.71 to 6.51)	-0.21 (-0.86 to 0.45)

Colombia	105421<br>(76835 to 145329)	701<br>(510.64 to 966.99)	72449<br>(51389 to 99990)	505.25<br>(357.81 to 698.3)	-1.17<br>(-1.44 to -0.91)	775<br>(223 to 1585)	5.15<br>(1.48 to 10.54)	533<br>(150 to 1105)	3.71<br>(1.04 to 7.68)	-1.17<br>(-1.43 to -0.91)
Comoros	1036<br>(742 to 1401)	391.91<br>(279.36 to 533.34)	1127<br>(788 to 1561)	358.22<br>(250.9 to 495.34)	-0.18<br>(-0.33 to -0.04)	8<br>(2 to 16)	2.88<br>(0.86 to 6.11)	8<br>(2 to 18)	2.63<br>(0.78 to 5.65)	-0.18<br>(-0.33 to -0.04)
Congo	4954<br>(3490 to 6775)	373.49<br>(262.13 to 512.86)	8022<br>(5493 to 11232)	321.97<br>(220.79 to 450.19)	0.16<br>(-5.04 to 5.63)	36<br>(11 to 78)	2.74<br>(0.83 to 5.91)	59<br>(17 to 128)	2.36<br>(0.7 to 5.14)	0.15<br>(-5.01 to 5.59)
Cook Islands	18<br>(12 to 25)	205.07<br>(142.99 to 285.31)	10<br>(7 to 14)	181.25<br>(123.84 to 257.67)	-0.42<br>(-0.79 to -0.06)	0<br>(0 to 0)	1.51<br>(0.44 to 3.25)	0<br>(0 to 0)	1.33<br>(0.39 to 2.96)	-0.42<br>(-0.77 to -0.06)
Costa Rica	7914<br>(5569 to 11093)	553.8<br>(389.39 to 776.41)	6893<br>(4773 to 9697)	510.85<br>(354.46 to 718.31)	-0.26<br>(-0.3 to -0.21)	58<br>(16 to 120)	4.07<br>(1.15 to 8.41)	51<br>(14 to 107)	3.76<br>(1.06 to 7.93)	-0.26<br>(-0.3 to -0.21)
Cote d'Ivoire	27061<br>(19461 to 36538)	389.88<br>(277.85 to 531.8)	51478<br>(36510 to 69939)	359.21<br>(253.46 to 491)	-0.23<br>(-0.61 to 0.15)	198<br>(60 to 420)	2.86<br>(0.86 to 6.1)	378<br>(113 to 815)	2.64<br>(0.79 to 5.7)	-0.23<br>(-0.6 to 0.15)
Croatia	15321<br>(10779 to 21531)	1132.95<br>(798.18 to 1589.77)	6928<br>(4970 to 9505)	846.33<br>(609.12 to 1158.48)	-1.56<br>(-2.04 to -1.07)	113<br>(32 to 233)	8.32<br>(2.35 to 17.27)	51<br>(14 to 105)	6.22<br>(1.77 to 12.82)	-1.55<br>(-2.03 to -1.07)
Cuba	21580<br>(15571 to 29115)	582.82<br>(420.5 to 784.12)	15384<br>(10869 to 20997)	637.26<br>(451.05 to 867)	0.28<br>(0.24 to 0.32)	159<br>(47 to 336)	4.28<br>(1.27 to 9.07)	113<br>(33 to 242)	4.68<br>(1.37 to 10.02)	0.28<br>(0.24 to 0.32)

Cyprus	2762 (1962 to 3804)	1062.97 (757.56 to 1460.46)	2892 (2025 to 4011)	1012.12 (709.45 to 1402)	-0.16 (-0.17 to -0.15)	20 (6 to 44)	7.81 (2.35 to 17.02)	21 (6 to 47)	7.44 (2.24 to 16.29)	-0.16 (-0.17 to -0.15)
Czechia	38968 (25939 to 58356)	1263.78 (843.69 to 1889.81)	20444 (13447 to 30674)	917.4 (604.71 to 1373.36)	-0.97 (-1.21 to -0.74)	286 (78 to 616)	9.29 (2.52 to 19.98)	150 (41 to 329)	6.74 (1.86 to 14.71)	-0.97 (-1.21 to -0.74)
Democratic People's Republic of Korea	16373 (11054 to 23706)	208.16 (139.7 to 302.41)	13080 (8292 to 19785)	190.27 (121.48 to 285.5)	-0.31 (-0.41 to -0.21)	120 (34 to 266)	1.53 (0.44 to 3.38)	96 (27 to 216)	1.4 (0.4 to 3.13)	-0.31 (-0.41 to -0.21)
Democratic Republic of the Congo	86962 (62415 to 117894)	399.09 (283.73 to 546.15)	170384 (120963 to 233305)	357.24 (253.15 to 490.54)	-0.28 (-0.83 to 0.27)	637 (197 to 1357)	2.93 (0.9 to 6.27)	1250 (378 to 2713)	2.62 (0.79 to 5.7)	-0.28 (-0.83 to 0.27)
Denmark	13134 (9244 to 18270)	1033.31 (731.74 to 1424.3)	13121 (9061 to 18377)	1005.5 (697.77 to 1400.15)	-0.08 (-0.1 to -0.07)	97 (29 to 211)	7.6 (2.29 to 16.5)	96 (29 to 212)	7.39 (2.22 to 16.2)	-0.08 (-0.1 to -0.07)
Djibouti	1016 (730 to 1380)	453.39 (325.05 to 616.19)	1937 (1379 to 2647)	366.99 (261.13 to 502.03)	-0.8 (-1.15 to -0.45)	7 (2 to 16)	3.33 (1.02 to 7.06)	14 (4 to 31)	2.69 (0.8 to 5.79)	-0.8 (-1.14 to -0.45)
Dominica	169 (120 to 231)	514.28 (364.54 to 702.3)	102 (72 to 140)	511.49 (360.16 to 699.22)	-0.1 (-0.3 to 0.1)	1 (0 to 3)	3.78 (1.13 to 8.08)	1 (0 to 2)	3.76 (1.1 to 8.03)	-0.1 (-0.3 to 0.1)
Dominican Republic	18049 (12984 to 24317)	515.92 (370.69 to 695.47)	20647 (14643 to 28106)	528.03 (374.32 to 718.45)	0.07 (-0.04 to 0.19)	133 (40 to 281)	3.79 (1.14 to 8.04)	152 (45 to 321)	3.88 (1.15 to 8.2)	0.08 (-0.04 to 0.19)

Ecuador	27924 (20354 to 37612)	563.44 (410.71 to 759)	33644 (23879 to 45884)	507.14 (360.37 to 690.65)	-0.34 (-0.41 to -0.26)	205 (62 to 435)	4.14 (1.24 to 8.77)	247 (74 to 533)	3.73 (1.11 to 8.02)	-0.34 (-0.41 to -0.26)
Egypt	114280 (84701 to 151191)	413.35 (305.43 to 548.72)	154894 (110005 to 211776)	335.85 (238.37 to 459.96)	-0.63 (-0.73 to -0.53)	839 (256 to 1794)	3.03 (0.93 to 6.5)	1138 (341 to 2468)	2.47 (0.74 to 5.36)	-0.63 (-0.73 to -0.53)
El Salvador	20023 (13934 to 29750)	730.87 (507.57 to 1088.91)	11549 (8123 to 16048)	485.32 (341.4 to 674.37)	-1.34 (-1.62 to -1.07)	147 (42 to 318)	5.37 (1.54 to 11.62)	85 (24 to 180)	3.57 (1.01 to 7.57)	-1.34 (-1.61 to -1.07)
Equatorial Guinea	997 (720 to 1349)	412.21 (295.29 to 563.12)	2560 (1739 to 3616)	330.21 (224.98 to 464.35)	-0.71 (-0.83 to -0.6)	7 (2 to 15)	3.02 (0.92 to 6.37)	19 (6 to 41)	2.42 (0.72 to 5.25)	-0.71 (-0.83 to -0.59)
Eritrea	22940 (12616 to 43582)	1203.75 (660.51 to 2290.84)	11638 (8245 to 15826)	362.56 (256.41 to 494.06)	-4.44 (-7.4 to -1.39)	167 (46 to 407)	8.77 (2.42 to 21.34)	85 (26 to 184)	2.66 (0.8 to 5.74)	-4.42 (-7.33 to -1.41)
Estonia	3938 (2843 to 5523)	852.76 (615.28 to 1197.26)	1719 (1205 to 2429)	606.61 (425.66 to 854.74)	-1.1 (-1.12 to -1.07)	29 (8 to 59)	6.27 (1.76 to 12.87)	13 (4 to 26)	4.46 (1.26 to 9.28)	-1.1 (-1.12 to -1.07)
Eswatini	1807 (1298 to 2426)	382.43 (274.05 to 516.08)	2081 (1475 to 2820)	387.75 (274.99 to 525.35)	0 (-0.06 to 0.07)	13 (4 to 28)	2.81 (0.83 to 5.96)	15 (5 to 33)	2.84 (0.87 to 6.06)	0 (-0.06 to 0.06)
Ethiopia	252043 (164053 to 400700)	898.18 (577.06 to 1445.55)	270381 (188737 to 377232)	472.88 (329.89 to 659.93)	-2.23 (-4.06 to -0.35)	1845 (540 to 4068)	6.57 (1.93 to 14.54)	1982 (599 to 4282)	3.47 (1.05 to 7.49)	-2.22 (-4.03 to -0.37)

Fiji	698 (492 to 971)	195.17 (137.49 to 271.53)	623 (435 to 879)	177.34 (123.88 to 249.89)	-0.37 (-0.44 to -0.31)	5 (1 to 11)	1.44 (0.42 to 3.1)	5 (1 to 10)	1.3 (0.38 to 2.84)	-0.37 (-0.44 to -0.31)
Finland	21601 (14689 to 30725)	1691.9 (1155.32 to 2399.08)	19078 (12705 to 27484)	1637.66 (1102.44 to 2342.04)	-0.1 (-0.16 to -0.05)	159 (47 to 351)	12.43 (3.72 to 27.39)	140 (41 to 313)	12.03 (3.57 to 26.69)	-0.1 (-0.16 to -0.04)
France	177153 (126362 to 243508)	1082.12 (774.33 to 1478.79)	163264 (113492 to 226800)	1026.48 (717.9 to 1417.2)	-0.17 (-0.19 to -0.15)	1302 (392 to 2828)	7.96 (2.39 to 17.28)	1200 (362 to 2615)	7.55 (2.28 to 16.38)	-0.17 (-0.19 to -0.15)
Gabon	1951 (1381 to 2644)	383.47 (270.14 to 522.93)	2761 (1911 to 3851)	333.45 (231.11 to 464.58)	-0.43 (-0.52 to -0.35)	14 (4 to 31)	2.81 (0.85 to 6.02)	20 (6 to 44)	2.45 (0.72 to 5.29)	-0.43 (-0.52 to -0.35)
Gambia	2230 (1593 to 3012)	393.64 (278.64 to 537.33)	4421 (3093 to 6075)	349.2 (243.91 to 480.8)	-0.38 (-0.52 to -0.25)	16 (5 to 35)	2.89 (0.86 to 6.17)	32 (10 to 70)	2.56 (0.76 to 5.5)	-0.38 (-0.52 to -0.25)
Georgia	13491 (9840 to 18170)	745.95 (544.25 to 1003.76)	6515 (4851 to 8529)	705.49 (525.97 to 922.75)	-0.05 (-0.98 to 0.9)	99 (29 to 204)	5.48 (1.6 to 11.27)	48 (14 to 101)	5.18 (1.51 to 10.9)	-0.05 (-0.98 to 0.89)
Germany	181204 (128270 to 249826)	1039.23 (737.33 to 1428.34)	161550 (112088 to 225207)	1008.47 (702.06 to 1400.87)	-0.1 (-0.11 to -0.09)	1332 (404 to 2906)	7.64 (2.31 to 16.67)	1188 (358 to 2605)	7.41 (2.23 to 16.24)	-0.1 (-0.11 to -0.09)
Ghana	30443 (21404 to 41541)	368.69 (257.45 to 507.28)	56022 (39073 to 77682)	344 (239.44 to 478.39)	-0.22 (-0.29 to -0.15)	223 (67 to 473)	2.7 (0.81 to 5.77)	411 (122 to 882)	2.52 (0.75 to 5.42)	-0.22 (-0.28 to -0.15)

Greece	30203 (21206 to 41749)	1059 (749.76 to 1450.9)	19163 (13268 to 26804)	993.57 (692.5 to 1379.99)	-0.2 (-0.22 to -0.18)	222 (67 to 485)	7.79 (2.36 to 16.92)	141 (42 to 309)	7.3 (2.2 to 15.95)	-0.2 (-0.22 to -0.18)
Greenland	133 (98 to 176)	749.71 (552.78 to 996.97)	92 (65 to 126)	596.95 (422.07 to 816.41)	-0.74 (-0.84 to -0.64)	1 (0 to 2)	5.51 (1.64 to 11.43)	1 (0 to 1)	4.39 (1.31 to 9.3)	-0.74 (-0.84 to -0.64)
Grenada	239 (171 to 323)	569.22 (408.29 to 771.89)	183 (131 to 247)	590.9 (422.94 to 796.1)	0.06 (-0.1 to 0.22)	2 (1 to 4)	4.18 (1.23 to 8.75)	1 (0 to 3)	4.34 (1.29 to 9.08)	0.06 (-0.1 to 0.22)
Guam	114 (80 to 157)	209.95 (147.65 to 291.47)	88 (61 to 125)	178.69 (123.51 to 253.44)	-0.54 (-0.58 to -0.49)	1 (0 to 2)	1.54 (0.45 to 3.34)	1 (0 to 1)	1.31 (0.38 to 2.89)	-0.54 (-0.58 to -0.49)
Guatemala	38044 (27920 to 52667)	763.53 (556.97 to 1067.51)	41007 (29932 to 56184)	620.33 (453.15 to 849.12)	-0.66 (-0.85 to -0.46)	279 (81 to 578)	5.6 (1.62 to 11.69)	301 (88 to 621)	4.55 (1.32 to 9.38)	-0.65 (-0.85 to -0.46)
Guinea-Bissau	2506 (1833 to 3344)	423.26 (307.44 to 569.75)	3900 (2772 to 5278)	350.56 (248.08 to 476.91)	-0.81 (-1.15 to -0.47)	18 (6 to 39)	3.1 (0.95 to 6.61)	29 (9 to 62)	2.57 (0.77 to 5.56)	-0.81 (-1.15 to -0.47)
Guinea	14441 (10439 to 19254)	430.04 (307.29 to 581.28)	28271 (20339 to 37977)	379 (271.36 to 512.58)	-0.39 (-0.53 to -0.24)	106 (32 to 222)	3.15 (0.96 to 6.69)	207 (64 to 444)	2.78 (0.85 to 5.98)	-0.39 (-0.53 to -0.24)
Guyana	2133 (1548 to 2852)	557.24 (403.39 to 746.36)	1567 (1134 to 2108)	556.54 (402.78 to 748.28)	-0.04 (-0.08 to -0.01)	16 (5 to 33)	4.09 (1.25 to 8.67)	12 (3 to 24)	4.09 (1.23 to 8.66)	-0.04 (-0.08 to -0.01)

Haiti	20860 (15284 to 27706)	629.3 (459.9 to 838.2)	35951 (26578 to 47962)	641.62 (474.12 to 856.53)	0.03 (-0.48 to 0.53)	153 (46 to 318)	4.62 (1.37 to 9.6)	264 (79 to 549)	4.71 (1.41 to 9.8)	0.02 (-0.46 to 0.51)
Honduras	17022 (12505 to 23427)	619.19 (454.56 to 852.13)	22065 (15966 to 30248)	509.84 (368.82 to 699.14)	-0.71 (-0.91 to -0.51)	125 (35 to 260)	4.55 (1.28 to 9.45)	162 (46 to 336)	3.75 (1.06 to 7.77)	-0.71 (-0.9 to -0.52)
Hungary	29365 (19603 to 43293)	998.75 (668.83 to 1468.13)	16589 (11107 to 24501)	878.23 (588.87 to 1295.24)	-0.41 (-0.45 to -0.38)	216 (60 to 467)	7.34 (2.03 to 15.85)	122 (33 to 264)	6.46 (1.77 to 13.96)	-0.41 (-0.44 to -0.38)
Iceland	929 (658 to 1268)	1091.63 (775.41 to 1485.19)	907 (629 to 1263)	1012.91 (704.57 to 1405.84)	-0.24 (-0.29 to -0.19)	7 (2 to 15)	8.02 (2.42 to 17.32)	7 (2 to 15)	7.45 (2.24 to 16.27)	-0.24 (-0.29 to -0.19)
India	1696405 (1195452 to 2358464)	417.61 (293.47 to 582.68)	1771988 (1154357 to 2605157)	343.17 (225.42 to 500.23)	-0.6 (-0.71 to -0.49)	12461 (3774 to 27308)	3.07 (0.93 to 6.73)	13017 (3775 to 29098)	2.52 (0.73 to 5.62)	-0.6 (-0.71 to -0.49)
Indonesia	253624 (184042 to 342370)	289.94 (210.67 to 390.87)	206863 (147517 to 282264)	226.94 (162.33 to 308.69)	-0.75 (-0.88 to -0.61)	1864 (543 to 3956)	2.13 (0.62 to 4.52)	1521 (439 to 3281)	1.67 (0.48 to 3.59)	-0.82 (-0.99 to -0.65)
Iran (Islamic Republic of)	172207 (126273 to 229691)	553.88 (406.08 to 740.04)	110729 (77853 to 153989)	424.89 (299.4 to 589.88)	-0.83 (-1 to -0.65)	1263 (382 to 2679)	4.06 (1.23 to 8.62)	813 (243 to 1758)	3.12 (0.93 to 6.74)	-0.82 (-0.99 to -0.65)
Iraq	66189 (48920 to 87449)	657.9 (486.04 to 870.06)	85248 (62603 to 113711)	475.53 (349.61 to 633.58)	-1.15 (-3.86 to 1.65)	486 (146 to 1008)	4.83 (1.45 to 10.02)	626 (187 to 1317)	3.49 (1.04 to 7.34)	-1.14 (-3.84 to 1.63)

Ireland	14974 (10650 to 20531)	1120.08 (801.27 to 1525.56)	13677 (9512 to 19085)	1029.73 (720.19 to 1429.45)	-0.27 (-0.28 to -0.26)	110 (33 to 238)	8.23 (2.47 to 17.73)	101 (30 to 221)	7.57 (2.27 to 16.55)	-0.27 (-0.28 to -0.25)
Israel	23224 (16829 to 31786)	1154.68 (838 to 1577.18)	34773 (24626 to 48056)	1031.61 (730.46 to 1426)	-0.34 (-0.45 to -0.22)	171 (51 to 370)	8.49 (2.56 to 18.37)	256 (77 to 559)	7.59 (2.29 to 16.59)	-0.33 (-0.45 to -0.22)
Italy	357807 (209526 to 584995)	2482.63 (1473.57 to 4030.32)	184928 (123720 to 263853)	1744.58 (1178.1 to 2477.7)	-1.13 (-1.16 to -1.09)	2617 (731 to 6051)	18.17 (5.08 to 41.81)	1356 (402 to 2998)	12.8 (3.79 to 28.2)	-1.12 (-1.16 to -1.08)
Jamaica	7265 (5234 to 9797)	665.87 (480.39 to 896.63)	4873 (3486 to 6622)	589.33 (422.07 to 798.04)	-0.41 (-0.57 to -0.25)	53 (16 to 112)	4.89 (1.45 to 10.26)	36 (11 to 76)	4.33 (1.31 to 9.17)	-0.41 (-0.57 to -0.25)
Japan	329362 (237871 to 441384)	968.08 (703.9 to 1285.48)	184137 (131225 to 248660)	853.26 (611.22 to 1145.09)	-0.4 (-0.46 to -0.35)	2420 (702 to 5114)	7.11 (2.08 to 14.99)	1353 (390 to 2894)	6.27 (1.82 to 13.38)	-0.4 (-0.45 to -0.35)
Jordan	8365 (6085 to 11228)	401.12 (291.53 to 538.85)	17181 (12090 to 23761)	339.88 (240.15 to 467.65)	-0.51 (-0.64 to -0.39)	61 (19 to 131)	2.95 (0.89 to 6.29)	126 (37 to 277)	2.5 (0.74 to 5.46)	-0.51 (-0.63 to -0.39)
Kazakhstan	51704 (38137 to 70415)	781.81 (576.35 to 1065.49)	40332 (29190 to 54795)	609.6 (440.64 to 829.46)	-0.81 (-0.88 to -0.74)	380 (108 to 778)	5.75 (1.64 to 11.76)	296 (87 to 621)	4.48 (1.31 to 9.39)	-0.81 (-0.88 to -0.74)
Kenya	63654 (44368 to 88143)	464.11 (321.72 to 647.16)	102266 (69236 to 145435)	412.43 (280.32 to 583.94)	-0.38 (-0.56 to -0.21)	467 (140 to 997)	3.4 (1.02 to 7.3)	750 (220 to 1633)	3.03 (0.89 to 6.57)	-0.38 (-0.56 to -0.21)

Kiribati	63<br>(44 to 88)	177.49<br>(124.04 to 248.71)	83<br>(56 to 119)	155.69<br>(105.07 to 223.69)	-0.62<br>(-0.92 to -0.33)	0<br>(0 to 1)	1.31<br>(0.38 to 2.86)	1<br>(0 to 1)	1.15<br>(0.33 to 2.53)	-0.62<br>(-0.92 to -0.33)
Kuwait	4804<br>(3009 to 8049)	726.16<br>(452.03 to 1226.41)	4038<br>(2838 to 5517)	365.58<br>(257.64 to 498.53)	-2.53<br>(-3.75 to -1.3)	35<br>(10 to 79)	5.32<br>(1.52 to 12.03)	30<br>(9 to 65)	2.69<br>(0.79 to 5.91)	-2.52<br>(-3.72 to -1.3)
Kyrgyzstan	13150<br>(9627 to 17524)	632.54<br>(462.06 to 845.51)	14730<br>(10571 to 20035)	529.43<br>(379.63 to 720.76)	-0.59<br>(-0.66 to -0.51)	97<br>(28 to 202)	4.65<br>(1.34 to 9.75)	108<br>(32 to 229)	3.89<br>(1.13 to 8.22)	-0.59<br>(-0.66 to -0.51)
Lao People's Democratic Republic	6667<br>(4730 to 9796)	304.96<br>(214.61 to 454.58)	5280<br>(3853 to 7127)	176.11<br>(128.42 to 237.76)	-1.57<br>(-1.91 to -1.22)	49<br>(14 to 109)	2.23<br>(0.64 to 5.01)	39<br>(11 to 83)	1.29<br>(0.37 to 2.77)	-1.56<br>(-1.9 to -1.21)
Latvia	6381<br>(4622 to 8940)	844.04<br>(610.86 to 1184.45)	2359<br>(1668 to 3370)	603.13<br>(426.98 to 860.11)	-1.09<br>(-1.13 to -1.05)	47<br>(13 to 97)	6.2<br>(1.78 to 12.79)	17<br>(5 to 36)	4.44<br>(1.25 to 9.28)	-1.09<br>(-1.12 to -1.05)
Lebanon	8516<br>(5842 to 12651)	634.22<br>(434.45 to 944.93)	6110<br>(4390 to 8334)	362.51<br>(260.87 to 493.66)	-1.48<br>(-1.97 to -0.99)	62<br>(18 to 138)	4.65<br>(1.36 to 10.28)	45<br>(13 to 98)	2.66<br>(0.79 to 5.82)	-1.48<br>(-1.97 to -0.99)
Lesotho	3055<br>(2190 to 4154)	368.48<br>(263.52 to 503.33)	3045<br>(2192 to 4145)	358.12<br>(258.29 to 485.73)	-0.07<br>(-0.18 to 0.04)	22<br>(7 to 48)	2.7<br>(0.81 to 5.8)	22<br>(7 to 48)	2.63<br>(0.78 to 5.65)	-0.07<br>(-0.18 to 0.05)
Liberia	11976<br>(7399 to 20930)	911.9<br>(554.06 to 1614.36)	9213<br>(6373 to 12742)	330.52<br>(228.45 to 457.62)	-2.11<br>(-8.17 to 4.34)	87<br>(26 to 203)	6.66<br>(1.95 to 15.51)	68<br>(20 to 147)	2.43<br>(0.71 to 5.29)	-2.1<br>(-8.12 to 4.3)

Libya	9217<br>(6655 to 12338)	400.23<br>(288.89 to 536.18)	8553<br>(6123 to 11708)	409.23<br>(294.37 to 556.11)	1.38<br>(0.57 to 2.19)	68<br>(20 to 146)	2.94<br>(0.88 to 6.34)	63<br>(19 to 138)	3.01<br>(0.89 to 6.6)	1.37<br>(0.58 to 2.18)
Lithuania	8294<br>(5967 to 11670)	743.08<br>(533.94 to 1047.56)	3211<br>(2236 to 4566)	593.73<br>(413.98 to 843.46)	-0.72<br>(-0.76 to -0.68)	61<br>(17 to 126)	5.46<br>(1.56 to 11.27)	24<br>(7 to 49)	4.36<br>(1.23 to 9.13)	-0.72<br>(-0.76 to -0.68)
Luxembourg	995<br>(715 to 1353)	1118.04<br>(803.58 to 1516.72)	1383<br>(961 to 1918)	1017.14<br>(709.43 to 1405.6)	-0.31<br>(-0.34 to -0.27)	7<br>(2 to 16)	8.22<br>(2.48 to 17.77)	10<br>(3 to 22)	7.48<br>(2.25 to 16.35)	-0.31<br>(-0.34 to -0.27)
Madagascar	26852<br>(19394 to 35987)	400.56<br>(287.68 to 540.22)	51880<br>(36389 to 72154)	347.83<br>(243.84 to 484.27)	-0.45<br>(-0.53 to -0.36)	197<br>(59 to 417)	2.94<br>(0.88 to 6.24)	381<br>(113 to 823)	2.55<br>(0.76 to 5.52)	-0.45<br>(-0.54 to -0.37)
Malawi	22070<br>(15905 to 29676)	394.12<br>(281.27 to 535.21)	35709<br>(24735 to 49749)	335.95<br>(233.14 to 467.05)	-0.53<br>(-0.61 to -0.44)	162<br>(49 to 343)	2.89<br>(0.87 to 6.17)	262<br>(78 to 567)	2.47<br>(0.73 to 5.33)	-0.52<br>(-0.61 to -0.44)
Malaysia	17520<br>(12547 to 24023)	211.19<br>(151.17 to 289.64)	19293<br>(13728 to 26722)	185.12<br>(131.87 to 256.16)	-0.44<br>(-0.5 to -0.38)	129<br>(37 to 274)	1.55<br>(0.45 to 3.31)	142<br>(41 to 306)	1.36<br>(0.39 to 2.93)	-0.44<br>(-0.5 to -0.38)
Maldives	327<br>(236 to 455)	260.43<br>(187.18 to 362.71)	253<br>(181 to 346)	192.6<br>(137.92 to 262.94)	-0.9<br>(-1.15 to -0.65)	2<br>(1 to 5)	1.91<br>(0.54 to 4.02)	2<br>(1 to 4)	1.42<br>(0.41 to 3.01)	-0.9<br>(-1.14 to -0.65)
Mali	22597<br>(16604 to 29941)	455.15<br>(330.46 to 609.03)	59241<br>(43362 to 79177)	416.54<br>(302.71 to 561)	-0.13<br>(-1.06 to 0.8)	166<br>(52 to 347)	3.34<br>(1.03 to 7.06)	435<br>(134 to 919)	3.06<br>(0.94 to 6.5)	-0.13<br>(-1.06 to 0.8)

Malta	958<br>(702 to 1270)	830.42<br>(610.12 to 1098.65)	660<br>(479 to 876)	784.44<br>(569.41 to 1040.55)	-0.18<br>(-0.21 to -0.16)	7<br>(2 to 15)	6.11<br>(1.81 to 12.86)	5<br>(1 to 10)	5.77<br>(1.7 to 12.24)	-0.18<br>(-0.21 to -0.16)
Marshall Islands	51<br>(36 to 71)	196.32<br>(138.47 to 272.46)	41<br>(28 to 58)	173.82<br>(120.66 to 246.08)	-0.39<br>(-0.42 to -0.36)	0<br>(0 to 1)	1.44<br>(0.43 to 3.14)	0<br>(0 to 1)	1.28<br>(0.37 to 2.8)	-0.39<br>(-0.43 to -0.36)
Mauritania	4434<br>(3182 to 5988)	390.22<br>(278.14 to 531.78)	7876<br>(5466 to 10902)	338.53<br>(234.47 to 470.07)	-0.41<br>(-0.54 to -0.28)	33<br>(10 to 69)	2.86<br>(0.86 to 6.08)	58<br>(17 to 125)	2.48<br>(0.74 to 5.36)	-0.41<br>(-0.54 to -0.28)
Mauritius	914<br>(658 to 1253)	211.09<br>(152.37 to 289.01)	543<br>(382 to 760)	177.97<br>(125.67 to 247.39)	-0.55<br>(-0.61 to -0.48)	7<br>(2 to 14)	1.55<br>(0.45 to 3.29)	4<br>(1 to 9)	1.31<br>(0.38 to 2.81)	-0.54<br>(-0.61 to -0.48)
Mexico	317882<br>(220768 to 455588)	733.08<br>(509.08 to 1050.47)	234679<br>(154905 to 353303)	536.85<br>(355.1 to 806.07)	-1.03<br>(-1.77 to -0.28)	2337<br>(671 to 5083)	5.39<br>(1.55 to 11.72)	1725<br>(493 to 3887)	3.95<br>(1.13 to 8.86)	-1.03<br>(-1.77 to -0.28)
Micronesia (Federated States of)	113<br>(80 to 157)	199.08<br>(141.17 to 276.32)	75<br>(52 to 107)	174.94<br>(121.79 to 247.25)	-0.45<br>(-0.68 to -0.22)	1<br>(0 to 2)	1.46<br>(0.43 to 3.17)	1<br>(0 to 1)	1.29<br>(0.37 to 2.83)	-0.45<br>(-0.67 to -0.22)
Monaco	51<br>(36 to 71)	1044.12<br>(734.47 to 1442.73)	68<br>(47 to 95)	997.31<br>(694.65 to 1385.6)	-0.15<br>(-0.16 to -0.14)	0<br>(0 to 1)	7.68<br>(2.31 to 16.8)	1<br>(0 to 1)	7.33<br>(2.2 to 16.03)	-0.15<br>(-0.16 to -0.14)
Mongolia	7754<br>(5720 to 10390)	691.87<br>(509.52 to 928.81)	8296<br>(6097 to 11188)	654.31<br>(480.21 to 883.48)	-0.22<br>(-0.37 to -0.07)	57<br>(17 to 118)	5.08<br>(1.48 to 10.55)	61<br>(18 to 127)	4.81<br>(1.4 to 9.99)	-0.22<br>(-0.37 to -0.07)

Montenegro	2119 (1428 to 3103)	982.31 (663.08 to 1437.6)	1323 (885 to 1941)	870.74 (582.25 to 1276.44)	-0.38 (-0.41 to -0.36)	16 (4 to 34)	7.22 (1.98 to 15.52)	10 (3 to 21)	6.4 (1.73 to 13.88)	-0.38 (-0.41 to -0.36)
Morocco	55613 (41214 to 72685)	447.9 (331.57 to 586.04)	48085 (34421 to 64917)	367.19 (263.16 to 495.01)	-0.65 (-0.69 to -0.62)	408 (123 to 864)	3.29 (0.99 to 6.96)	353 (105 to 759)	2.7 (0.8 to 5.79)	-0.65 (-0.69 to -0.62)
Mozambique	34569 (24961 to 47679)	464.35 (331.73 to 648.31)	63302 (45210 to 85940)	359.85 (256.35 to 490.67)	-0.84 (-1.14 to -0.53)	253 (77 to 544)	3.4 (1.03 to 7.37)	465 (140 to 1004)	2.64 (0.8 to 5.73)	-0.83 (-1.13 to -0.53)
Myanmar	54422 (40793 to 71741)	284.08 (213.02 to 374.34)	63187 (44391 to 90785)	297.72 (209.36 to 426.86)	-0.48 (-1.22 to 0.26)	400 (119 to 834)	2.09 (0.62 to 4.35)	464 (137 to 982)	2.18 (0.65 to 4.63)	-0.48 (-1.21 to 0.25)
Namibia	2822 (2020 to 3830)	371.3 (265.13 to 505.36)	3721 (2610 to 5123)	346.23 (243.09 to 476.08)	-0.24 (-0.51 to 0.03)	21 (6 to 44)	2.72 (0.82 to 5.81)	27 (8 to 59)	2.54 (0.76 to 5.44)	-0.24 (-0.51 to 0.03)
Nauru	10 (7 to 14)	198.54 (140.25 to 274.21)	9 (7 to 13)	184.28 (129.97 to 257.12)	-0.24 (-0.26 to -0.23)	0 (0 to 0)	1.46 (0.43 to 3.17)	0 (0 to 0)	1.36 (0.39 to 2.96)	-0.24 (-0.26 to -0.22)
Nepal	32746 (23179 to 44692)	321.28 (225.79 to 442.28)	37278 (25487 to 52440)	294.23 (202.01 to 411.8)	0.09 (-2.92 to 3.19)	241 (71 to 522)	2.36 (0.7 to 5.15)	274 (81 to 602)	2.16 (0.64 to 4.74)	0.08 (-2.89 to 3.15)
Netherlands	26497 (19458 to 35032)	688.62 (506.18 to 907.26)	24139 (17446 to 32239)	653.74 (473.46 to 869.01)	-0.18 (-0.39 to 0.04)	195 (58 to 407)	5.06 (1.5 to 10.58)	177 (52 to 374)	4.81 (1.42 to 10.1)	-0.18 (-0.39 to 0.04)

New Zealand	21388 (14838 to 30257)	1929.12 (1338.6 to 2728.73)	22962 (16488 to 31275)	1768.97 (1271.03 to 2406.66)	-0.29 (-0.33 to -0.24)	157 (44 to 330)	14.16 (3.99 to 29.72)	169 (48 to 343)	12.98 (3.67 to 26.39)	-0.29 (-0.33 to -0.24)
Nicaragua	13138 (9507 to 18170)	578.07 (418.34 to 799.2)	12591 (8890 to 17483)	485.62 (343.08 to 674.15)	-0.47 (-0.6 to -0.34)	97 (27 to 199)	4.25 (1.19 to 8.78)	93 (26 to 191)	3.57 (1.01 to 7.35)	-0.47 (-0.59 to -0.34)
Niger	21559 (15861 to 28864)	437.82 (318.37 to 593.35)	61472 (44461 to 82513)	396.47 (284.48 to 536.92)	-0.26 (-0.38 to -0.14)	158 (49 to 331)	3.21 (0.99 to 6.79)	451 (140 to 964)	2.91 (0.9 to 6.26)	-0.26 (-0.38 to -0.14)
Nigeria	226862 (159806 to 311551)	467.42 (326.38 to 648.29)	529955 (366900 to 735450)	417.04 (287.75 to 581.4)	-0.37 (-0.58 to -0.15)	1664 (495 to 3556)	3.43 (1.02 to 7.36)	3889 (1168 to 8439)	3.06 (0.92 to 6.65)	-0.37 (-0.58 to -0.15)
Niue	2 (1 to 3)	198.61 (139.27 to 276.33)	1 (1 to 1)	184.23 (129.03 to 258.91)	-0.28 (-0.52 to -0.04)	0 (0 to 0)	1.46 (0.43 to 3.19)	0 (0 to 0)	1.36 (0.39 to 2.97)	-0.28 (-0.51 to -0.04)
North Macedonia	7134 (4935 to 10257)	1015.18 (703.28 to 1458.05)	3956 (2662 to 5800)	870.31 (586.61 to 1275.33)	-0.5 (-0.62 to -0.38)	52 (15 to 111)	7.46 (2.08 to 15.77)	29 (8 to 63)	6.4 (1.76 to 13.77)	-0.52 (-0.62 to -0.41)
Northern Mariana Islands	36 (26 to 50)	221.25 (157.94 to 303.29)	31 (22 to 43)	199 (140.89 to 275.71)	-0.34 (-0.4 to -0.28)	0 (0 to 1)	1.63 (0.47 to 3.49)	0 (0 to 0)	1.46 (0.43 to 3.19)	-0.34 (-0.39 to -0.28)
Norway	15049 (10401 to 21005)	1328.99 (923.66 to 1842.38)	15319 (10390 to 21872)	1215.16 (829.39 to 1722.87)	-0.29 (-0.33 to -0.25)	111 (33 to 238)	9.76 (2.9 to 21.04)	113 (33 to 245)	8.93 (2.63 to 19.41)	-0.29 (-0.33 to -0.25)

Oman	4384 (3215 to 5828)	446.96 (326.55 to 597.69)	5408 (3883 to 7282)	369.82 (265.24 to 498.95)	-0.61 (-0.64 to -0.58)	32 (10 to 68)	3.28 (0.97 to 6.97)	40 (12 to 86)	2.72 (0.81 to 5.88)	-0.61 (-0.63 to -0.58)
Pakistan	218884 (147017 to 311749)	364.71 (243.48 to 523.43)	373971 (247215 to 542974)	343.34 (226.86 to 498.79)	-0.19 (-0.57 to 0.2)	1607 (475 to 3548)	2.68 (0.79 to 5.93)	2746 (810 to 6068)	2.52 (0.74 to 5.57)	-0.19 (-0.56 to 0.19)
Palau	15 (11 to 20)	236.4 (172.33 to 318.92)	10 (7 to 13)	209.17 (151.17 to 287.61)	-0.39 (-0.41 to -0.38)	0 (0 to 0)	1.74 (0.51 to 3.69)	0 (0 to 0)	1.54 (0.45 to 3.31)	-0.39 (-0.41 to -0.38)
Palestine	6015 (4318 to 8577)	524.83 (374.31 to 754.78)	9428 (6818 to 12644)	389.29 (281.81 to 521.64)	-1.12 (-4.69 to 2.59)	44 (13 to 96)	3.85 (1.14 to 8.36)	69 (21 to 150)	2.86 (0.87 to 6.19)	-1.11 (-4.66 to 2.57)
Panama	6070 (4345 to 8390)	560.58 (401.43 to 774.84)	7672 (5393 to 10735)	511.21 (359.95 to 714.25)	-0.29 (-0.34 to -0.23)	45 (13 to 92)	4.12 (1.17 to 8.51)	56 (16 to 118)	3.76 (1.05 to 7.84)	-0.29 (-0.34 to -0.23)
Papua New Guinea	4076 (2901 to 5561)	194.81 (138.12 to 266.77)	9448 (6816 to 12872)	197.17 (141.78 to 269.75)	-0.3 (-0.61 to 0.01)	30 (9 to 66)	1.43 (0.42 to 3.17)	69 (21 to 152)	1.45 (0.43 to 3.19)	-0.29 (-0.59 to 0.01)
Paraguay	13449 (9288 to 19044)	658.6 (454.41 to 934.55)	16621 (11113 to 23933)	613.73 (410.63 to 882.75)	-0.23 (-0.25 to -0.2)	99 (29 to 218)	4.85 (1.44 to 10.69)	122 (36 to 273)	4.51 (1.33 to 10.08)	-0.23 (-0.25 to -0.2)
Peru	75749 (55460 to 102968)	711.87 (521 to 968.26)	65168 (46226 to 88660)	519.31 (368.46 to 706.01)	-1.11 (-1.27 to -0.95)	556 (171 to 1152)	5.22 (1.61 to 10.83)	479 (144 to 1025)	3.81 (1.15 to 8.17)	-1.11 (-1.27 to -0.95)

Philippines	86590 (64166 to 113614)	273 (202.1 to 358.5)	94365 (66834 to 130672)	206.72 (146.56 to 285.81)	-0.93 (-1.54 to -0.31)	636 (191 to 1351)	2 (0.6 to 4.26)	694 (202 to 1516)	1.52 (0.44 to 3.32)	-0.92 (-1.53 to -0.31)
Poland	130247 (89517 to 189028)	1036.55 (714.06 to 1499.96)	62621 (42526 to 90931)	804.04 (546.36 to 1166.35)	-0.82 (-0.86 to -0.78)	957 (265 to 2033)	7.61 (2.11 to 16.15)	460 (127 to 989)	5.91 (1.64 to 12.69)	-0.82 (-0.85 to -0.79)
Portugal	30857 (22534 to 40966)	1015.39 (746.18 to 1339.04)	15693 (10941 to 21749)	811.57 (569.75 to 1114.2)	-0.72 (-0.74 to -0.7)	227 (66 to 485)	7.46 (2.19 to 15.93)	115 (35 to 248)	5.96 (1.8 to 12.79)	-0.72 (-0.74 to -0.7)
Puerto Rico	7805 (5545 to 10751)	584.1 (415.71 to 803.1)	3909 (2779 to 5336)	591.7 (422.51 to 802.81)	0.05 (-0.03 to 0.14)	57 (17 to 124)	4.29 (1.25 to 9.26)	29 (8 to 62)	4.35 (1.29 to 9.26)	0.05 (-0.03 to 0.13)
Qatar	615 (447 to 821)	411.19 (297.44 to 551.86)	2139 (1524 to 2903)	367.29 (260.82 to 500.76)	-0.37 (-0.41 to -0.32)	5 (1 to 10)	3.02 (0.91 to 6.48)	16 (5 to 34)	2.7 (0.8 to 5.85)	-0.37 (-0.41 to -0.33)
Republic of Korea	149151 (110443 to 196727)	920.99 (684.48 to 1209.18)	63349 (45704 to 85675)	747.24 (542.12 to 1003.12)	-0.68 (-0.72 to -0.64)	1096 (321 to 2272)	6.77 (1.99 to 14)	466 (135 to 984)	5.49 (1.61 to 11.53)	-0.68 (-0.72 to -0.64)
Republic of Moldova	12289 (9055 to 16877)	778.04 (573.34 to 1068.22)	3925 (2805 to 5418)	553.32 (396.52 to 761.23)	-1.09 (-1.15 to -1.03)	90 (27 to 186)	5.72 (1.69 to 11.78)	29 (8 to 60)	4.07 (1.17 to 8.43)	-1.09 (-1.15 to -1.03)
Romania	81555 (56378 to 117281)	1076.15 (744.91 to 1544.03)	35157 (23733 to 51269)	856.6 (579.85 to 1246.21)	-0.74 (-0.77 to -0.71)	599 (168 to 1250)	7.91 (2.22 to 16.48)	259 (69 to 554)	6.3 (1.7 to 13.49)	-0.74 (-0.77 to -0.71)

Russian Federation	451847 (322102 to 632947)	992.9 (708.31 to 1389.98)	201827 (145498 to 274502)	587.73 (424.68 to 797.13)	-1.7 (-1.92 to -1.49)	3320 (935 to 6820)	7.3 (2.06 to 14.98)	1484 (422 to 3102)	4.32 (1.23 to 9.02)	-1.7 (-1.92 to -1.49)
Rwanda	22610 (15727 to 33802)	567.85 (389.77 to 861.86)	21653 (15039 to 29949)	336.05 (233.43 to 464.56)	-2.3 (-3.53 to -1.06)	166 (50 to 366)	4.16 (1.25 to 9.29)	159 (47 to 344)	2.47 (0.73 to 5.34)	-2.29 (-3.5 to -1.06)
Saint Kitts and Nevis	111 (80 to 151)	603.31 (434.94 to 815.1)	97 (70 to 131)	665.74 (478.34 to 897.72)	0.33 (0.19 to 0.47)	1 (0 to 2)	4.43 (1.31 to 9.28)	1 (0 to 2)	4.89 (1.44 to 10.28)	0.33 (0.2 to 0.47)
Saint Lucia	361 (257 to 489)	539.24 (383.96 to 731.22)	239 (169 to 328)	558.84 (395.59 to 764.79)	0.11 (0.03 to 0.19)	3 (1 to 6)	3.96 (1.19 to 8.38)	2 (1 to 4)	4.11 (1.2 to 8.51)	0.11 (0.03 to 0.19)
Saint Vincent and the Grenadines	305 (220 to 413)	565.61 (409.8 to 763.08)	191 (137 to 259)	555.55 (398.76 to 751.47)	-0.12 (-0.2 to -0.04)	2 (1 to 5)	4.16 (1.24 to 8.71)	1 (0 to 3)	4.08 (1.2 to 8.58)	-0.12 (-0.19 to -0.04)
Samoa	191 (134 to 263)	208.53 (146.33 to 287.54)	174 (120 to 247)	174.77 (120.37 to 248.54)	-0.4 (-0.87 to 0.07)	1 (0 to 3)	1.53 (0.45 to 3.29)	1 (0 to 3)	1.29 (0.37 to 2.83)	-0.4 (-0.86 to 0.06)
San Marino	65 (46 to 91)	1063.93 (753.06 to 1466.97)	66 (46 to 92)	1034.5 (724.63 to 1427.4)	-0.09 (-0.1 to -0.08)	0 (0 to 1)	7.82 (2.35 to 17.05)	0 (0 to 1)	7.61 (2.27 to 16.62)	-0.09 (-0.1 to -0.08)
Sao Tome and Principe	306 (220 to 409)	435 (311.76 to 583.69)	387 (274 to 524)	379.62 (269.33 to 512.08)	-0.46 (-0.5 to -0.41)	2 (1 to 5)	3.19 (0.97 to 6.72)	3 (1 to 6)	2.79 (0.84 to 5.94)	-0.46 (-0.5 to -0.41)

Saudi Arabia	49437 (36421 to 66133)	609.56 (448.7 to 815.26)	51161 (37037 to 68651)	493.8 (357.72 to 661.59)	-0.71 (-0.92 to -0.49)	363 (108 to 759)	4.48 (1.33 to 9.36)	376 (110 to 786)	3.63 (1.06 to 7.59)	-0.71 (-0.92 to -0.5)
Senegal	18028 (12957 to 24335)	402.4 (286.68 to 548.47)	28048 (19567 to 38648)	346.09 (241.11 to 477.6)	-0.53 (-0.6 to -0.46)	132 (40 to 281)	2.95 (0.9 to 6.31)	206 (61 to 443)	2.54 (0.76 to 5.47)	-0.53 (-0.6 to -0.46)
Serbia	29704 (20352 to 42833)	1011.8 (694.37 to 1456.84)	16523 (11051 to 24408)	859.54 (575.27 to 1268.21)	-0.68 (-0.98 to -0.38)	218 (61 to 466)	7.43 (2.09 to 15.85)	121 (33 to 262)	6.32 (1.74 to 13.63)	-0.68 (-0.98 to -0.38)
Seychelles	71 (51 to 98)	226.71 (162.83 to 310.23)	56 (40 to 78)	183.5 (130.76 to 254.29)	-0.64 (-0.7 to -0.58)	1 (0 to 1)	1.67 (0.47 to 3.54)	0 (0 to 1)	1.35 (0.39 to 2.9)	-0.64 (-0.7 to -0.58)
Sierra Leone	9427 (6915 to 12608)	415.71 (300.36 to 564.07)	16261 (11508 to 22252)	356.91 (251.59 to 490.14)	-0.9 (-3.63 to 1.9)	69 (21 to 146)	3.05 (0.92 to 6.48)	119 (36 to 259)	2.62 (0.78 to 5.69)	-0.9 (-3.62 to 1.89)
Singapore	7939 (5830 to 10594)	847.31 (623.88 to 1123.73)	8281 (6039 to 11082)	795.27 (579.96 to 1065.01)	-0.2 (-0.23 to -0.17)	58 (17 to 122)	6.23 (1.83 to 13.01)	61 (18 to 128)	5.84 (1.71 to 12.32)	-0.2 (-0.23 to -0.17)
Slovakia	18858 (12747 to 27784)	1060.19 (718.44 to 1560.51)	10246 (6838 to 15057)	907.02 (605.79 to 1332.19)	-0.51 (-0.56 to -0.46)	139 (38 to 298)	7.79 (2.15 to 16.73)	75 (21 to 161)	6.67 (1.82 to 14.25)	-0.51 (-0.56 to -0.46)
Slovenia	7360 (4911 to 10938)	1317.95 (882.62 to 1953.82)	4308 (2867 to 6337)	1057.35 (705.13 to 1550.11)	-0.73 (-0.8 to -0.65)	54 (15 to 115)	9.68 (2.7 to 20.55)	32 (9 to 69)	7.77 (2.16 to 16.8)	-0.73 (-0.8 to -0.65)

Solomon Islands	746<br>(529 to 1027)	195.45<br>(138.3 to 269.93)	1207<br>(860 to 1680)	184.55<br>(131.35 to 257.1)	-0.18<br>(-0.32 to -0.04)	3<br>(1 to 6)	1.44<br>(0.42 to 3.12)	4<br>(1 to 10)	1.36<br>(0.39 to 3)	-0.18<br>(-0.32 to -0.04)
Somalia	25971<br>(18057 to 38691)	570.91<br>(391.13 to 866.31)	52867<br>(38668 to 70796)	421.46<br>(306.46 to 567.35)	-1.24<br>(-2.64 to 0.17)	190<br>(57 to 418)	4.18<br>(1.25 to 9.27)	388<br>(120 to 825)	3.09<br>(0.95 to 6.6)	-1.24<br>(-2.62 to 0.17)
South Africa	85005<br>(60660 to 115785)	482.21<br>(343.93 to 656.84)	78615<br>(53235 to 111409)	391.33<br>(265.8 to 552.59)	-0.68<br>(-0.79 to -0.56)	624<br>(187 to 1327)	3.54<br>(1.06 to 7.53)	577<br>(170 to 1256)	2.87<br>(0.85 to 6.24)	-0.68<br>(-0.79 to -0.56)
South Sudan	13561<br>(9705 to 18289)	411.38<br>(292.87 to 557.42)	22691<br>(16433 to 30285)	414.58<br>(299.86 to 553.85)	0.44<br>(-3.23 to 4.26)	99<br>(30 to 211)	3.02<br>(0.91 to 6.42)	166<br>(51 to 353)	3.04<br>(0.93 to 6.46)	0.44<br>(-3.22 to 4.25)
Spain	119857<br>(84964 to 167225)	1046.62<br>(746.87 to 1443.82)	87717<br>(60428 to 123347)	984.4<br>(683.63 to 1374.13)	-0.2<br>(-0.24 to -0.16)	881<br>(267 to 1920)	7.69<br>(2.34 to 16.69)	645<br>(194 to 1418)	7.24<br>(2.18 to 15.82)	-0.2<br>(-0.24 to -0.16)
Sri Lanka	41688<br>(25005 to 74679)	562.35<br>(338.53 to 1003.55)	14335<br>(10397 to 19224)	202.41<br>(146.96 to 271.07)	-3.19<br>(-9.33 to 3.38)	305<br>(84 to 702)	4.11<br>(1.13 to 9.46)	105<br>(30 to 225)	1.49<br>(0.43 to 3.17)	-3.18<br>(-9.26 to 3.31)
Sudan	58440<br>(41479 to 84634)	542.85<br>(382.49 to 793.93)	76508<br>(55257 to 104509)	357.04<br>(258.02 to 487.52)	-1.37<br>(-2.44 to -0.29)	429<br>(128 to 938)	3.98<br>(1.19 to 8.73)	562<br>(169 to 1214)	2.62<br>(0.79 to 5.66)	-1.37<br>(-2.43 to -0.29)
Suriname	895<br>(635 to 1220)	521.79<br>(370.61 to 710.52)	1021<br>(729 to 1396)	533.81<br>(381.89 to 727.85)	0.07<br>(0.05 to 0.09)	7<br>(2 to 14)	3.83<br>(1.15 to 8.14)	8<br>(2 to 16)	3.92<br>(1.18 to 8.31)	0.07<br>(0.05 to 0.09)

Sweden	29349 (20335 to 40649)	1385.14 (962.52 to 1910.91)	30880 (21010 to 43722)	1271.26 (868.9 to 1792.18)	-0.27 (-0.34 to -0.21)	216 (64 to 465)	10.17 (3.01 to 21.96)	227 (67 to 495)	9.34 (2.77 to 20.35)	-0.27 (-0.34 to -0.21)
Switzerland	21892 (15665 to 29856)	1363.01 (977.67 to 1854.38)	20995 (14579 to 28852)	1183.47 (824.52 to 1622.18)	-0.46 (-0.5 to -0.42)	161 (47 to 339)	10.01 (2.95 to 21.12)	154 (46 to 332)	8.7 (2.58 to 18.72)	-0.46 (-0.5 to -0.42)
Syrian Arab Republic	27958 (20410 to 37446)	385.41 (280.71 to 517.71)	23521 (16699 to 32341)	406.72 (292.02 to 554.65)	0.32 (-0.56 to 1.2)	205 (62 to 440)	2.83 (0.85 to 6.07)	173 (52 to 376)	2.99 (0.91 to 6.5)	0.32 (-0.55 to 1.19)
Taiwan (Province of China)	16511 (11088 to 23867)	222.92 (151.19 to 319.2)	8069 (5055 to 12192)	194.82 (123.68 to 291.09)	-0.53 (-0.64 to -0.42)	121 (35 to 269)	1.64 (0.48 to 3.61)	59 (17 to 132)	1.43 (0.41 to 3.19)	-0.52 (-0.63 to -0.42)
Tajikistan	16738 (12247 to 22697)	599.23 (436.92 to 816.55)	22595 (16271 to 30926)	518.78 (372.82 to 711.69)	-1.37 (-2.05 to -0.69)	123 (36 to 258)	4.41 (1.27 to 9.28)	166 (48 to 352)	3.81 (1.1 to 8.08)	-1.37 (-2.04 to -0.69)
Thailand	60844 (44624 to 82433)	263.14 (193.09 to 356.41)	27523 (20120 to 36973)	193.1 (140.9 to 259.16)	-1.08 (-1.4 to -0.75)	447 (129 to 934)	1.93 (0.56 to 4.03)	202 (58 to 434)	1.42 (0.41 to 3.04)	-1.07 (-1.4 to -0.75)
Timor-Leste	1870 (1143 to 3248)	497.7 (298.73 to 879.74)	1237 (900 to 1674)	179.07 (130.2 to 242.29)	-4.7 (-5.93 to -3.45)	14 (4 to 32)	3.64 (1.02 to 8.58)	9 (3 to 19)	1.32 (0.38 to 2.81)	-4.68 (-5.9 to -3.44)
Togo	8541 (6082 to 11531)	395.19 (279.44 to 538.28)	14350 (10018 to 19651)	344.69 (240.22 to 473.4)	-0.46 (-0.56 to -0.35)	63 (19 to 133)	2.9 (0.88 to 6.19)	105 (31 to 228)	2.53 (0.75 to 5.49)	-0.46 (-0.56 to -0.35)

Tokelau	2<br>(1 to 2)	202.79<br>(141.6 to 282.2)	1<br>(1 to 1)	176.29<br>(120.74 to 249.97)	-0.44<br>(-0.5 to -0.38)	0<br>(0 to 0)	1.49<br>(0.44 to 3.26)	0<br>(0 to 0)	1.3<br>(0.37 to 2.86)	-0.44<br>(-0.5 to -0.37)
Tonga	104<br>(73 to 144)	195.37<br>(137.28 to 271.98)	82<br>(57 to 118)	167.69<br>(115.18 to 240.34)	-0.45<br>(-0.57 to -0.32)	1<br>(0 to 2)	1.44<br>(0.42 to 3.14)	1<br>(0 to 1)	1.23<br>(0.35 to 2.73)	-0.45<br>(-0.57 to -0.32)
Trinidad and Tobago	2741<br>(1955 to 3711)	527.11<br>(376.36 to 713.29)	1975<br>(1407 to 2674)	535.11<br>(382.04 to 723.42)	0.08<br>(-0.23 to 0.39)	20<br>(6 to 43)	3.88<br>(1.15 to 8.27)	15<br>(4 to 31)	3.93<br>(1.19 to 8.43)	0.08<br>(-0.23 to 0.39)
Tunisia	20248<br>(14636 to 26984)	507.37<br>(366.84 to 676.15)	14556<br>(10398 to 19611)	402.65<br>(288.03 to 541.79)	-0.76<br>(-0.81 to -0.71)	149<br>(44 to 310)	3.73<br>(1.09 to 7.77)	107<br>(32 to 227)	2.96<br>(0.88 to 6.27)	-0.76<br>(-0.81 to -0.71)
Turkey	116862<br>(85658 to 155904)	435.96<br>(319.92 to 580.88)	89506<br>(63459 to 123735)	353.86<br>(251.71 to 487.24)	-0.67<br>(-0.93 to -0.41)	859<br>(259 to 1840)	3.2<br>(0.97 to 6.86)	658<br>(193 to 1440)	2.6<br>(0.76 to 5.68)	-0.67<br>(-0.92 to -0.41)
Turkmenistan	12790<br>(9409 to 17286)	685.81<br>(503.28 to 929.36)	12049<br>(8672 to 16476)	616.14<br>(443.4 to 842.6)	-0.44<br>(-0.62 to -0.27)	94<br>(27 to 193)	5.04<br>(1.46 to 10.39)	88<br>(25 to 184)	4.52<br>(1.3 to 9.41)	-0.44<br>(-0.62 to -0.27)
Tuvalu	8<br>(6 to 11)	202.96<br>(144.79 to 278.96)	8<br>(6 to 12)	170.75<br>(118.05 to 242.6)	-0.59<br>(-0.67 to -0.51)	0<br>(0 to 0)	1.49<br>(0.44 to 3.24)	0<br>(0 to 0)	1.26<br>(0.36 to 2.76)	-0.59<br>(-0.67 to -0.51)
Uganda	42265<br>(30647 to 56589)	412.97<br>(296.7 to 557.3)	86495<br>(61190 to 118755)	348.31<br>(245.73 to 479.91)	-0.62<br>(-1.27 to 0.03)	310<br>(95 to 660)	3.03<br>(0.93 to 6.5)	635<br>(188 to 1377)	2.56<br>(0.76 to 5.55)	-0.62<br>(-1.27 to 0.03)

Ukraine	115131 (83925 to 156949)	757.05 (552.06 to 1031.66)	55044 (40048 to 74857)	622.68 (454.48 to 844.17)	-0.65 (-0.74 to -0.55)	846 (242 to 1749)	5.56 (1.6 to 11.49)	405 (115 to 840)	4.58 (1.3 to 9.48)	-0.65 (-0.74 to -0.55)
United Arab Emirates	3029 (2213 to 4024)	441.51 (321.56 to 589.18)	6152 (4340 to 8383)	364.28 (257.3 to 496.36)	-0.63 (-0.73 to -0.52)	22 (7 to 47)	3.24 (0.95 to 6.86)	45 (13 to 98)	2.68 (0.79 to 5.8)	-0.63 (-0.73 to -0.52)
United Kingdom	184433 (127505 to 258423)	1230.97 (854.42 to 1716.6)	181126 (122199 to 259126)	1144.25 (776.03 to 1628.05)	-0.24 (-0.25 to -0.22)	1355 (402 to 2929)	9.05 (2.69 to 19.55)	1331 (388 to 2921)	8.41 (2.46 to 18.41)	-0.24 (-0.25 to -0.22)
United Republic of Tanzania	60479 (43834 to 81155)	404.58 (291.01 to 546.6)	110784 (78687 to 151560)	359.8 (254.99 to 493.66)	-0.38 (-0.45 to -0.31)	444 (134 to 941)	2.97 (0.89 to 6.33)	813 (242 to 1755)	2.64 (0.79 to 5.71)	-0.38 (-0.45 to -0.31)
United States of America	689414 (478244 to 956128)	923.89 (640.99 to 1280.47)	608629 (392322 to 901791)	729.94 (472.22 to 1077.92)	-0.81 (-1.05 to -0.56)	5064 (1487 to 10881)	6.79 (1.99 to 14.59)	4469 (1292 to 9892)	5.36 (1.55 to 11.82)	-0.81 (-1.05 to -0.56)
United States Virgin Islands	244 (174 to 331)	583.28 (416.51 to 790.84)	102 (72 to 142)	558.38 (392.97 to 773.66)	-0.15 (-0.23 to -0.08)	2 (1 to 4)	4.29 (1.27 to 9.06)	1 (0 to 2)	4.1 (1.19 to 8.78)	-0.15 (-0.22 to -0.08)
Uruguay	15167 (10725 to 21012)	1392.88 (986.04 to 1928.24)	10414 (7508 to 14298)	1130.07 (814.42 to 1550.52)	-0.68 (-0.72 to -0.64)	111 (32 to 232)	10.22 (2.93 to 21.34)	76 (22 to 157)	8.3 (2.41 to 17.08)	-0.68 (-0.72 to -0.64)
Uzbekistan	65310 (47719 to 88270)	624.04 (454.88 to 846.4)	66941 (48140 to 91183)	542.44 (389.43 to 740.61)	-0.46 (-0.51 to -0.4)	480 (142 to 1005)	4.59 (1.35 to 9.62)	492 (143 to 1039)	3.99 (1.16 to 8.43)	-0.46 (-0.51 to -0.4)

Vanuatu	155 (109 to 214)	190.1 (132.67 to 264.76)	248 (171 to 353)	170.26 (117.28 to 242.54)	-0.41 (-0.56 to -0.25)	1 (0 to 2)	1.4 (0.41 to 3.05)	2 (1 to 4)	1.25 (0.36 to 2.75)	-0.41 (-0.56 to -0.25)
Venezuela (Bolivarian Republic of)	67255 (48068 to 94087)	740.88 (529.49 to 1036.67)	57903 (41043 to 81039)	661.85 (468.88 to 926.52)	-0.37 (-0.5 to -0.25)	494 (141 to 1018)	5.44 (1.56 to 11.21)	426 (119 to 882)	4.86 (1.36 to 10.07)	-0.37 (-0.49 to -0.25)
Viet Nam	85411 (62175 to 116487)	255.3 (185.83 to 348.36)	69824 (51221 to 94702)	221.15 (162.46 to 299.59)	-0.43 (-0.59 to -0.27)	627 (182 to 1298)	1.87 (0.54 to 3.88)	513 (147 to 1077)	1.63 (0.47 to 3.41)	-0.43 (-0.59 to -0.26)
Yemen	32570 (23818 to 43027)	394.66 (286.74 to 526.79)	110985 (71233 to 182424)	643.5 (412.13 to 1061.23)	1.5 (0.54 to 2.46)	239 (72 to 511)	2.9 (0.87 to 6.21)	813 (232 to 1873)	4.71 (1.35 to 10.87)	1.49 (0.54 to 2.45)
Zambia	18242 (13192 to 24527)	387.2 (278.08 to 523.83)	35417 (24789 to 48887)	340.09 (237.74 to 470.48)	-0.42 (-0.47 to -0.36)	134 (40 to 286)	2.84 (0.85 to 6.09)	260 (77 to 566)	2.5 (0.74 to 5.44)	-0.42 (-0.47 to -0.36)
Zimbabwe	21244 (14930 to 29303)	354.53 (248.57 to 490.75)	25868 (18052 to 35875)	325.23 (226.74 to 451.7)	-0.24 (-0.3 to -0.17)	156 (47 to 335)	2.6 (0.78 to 5.6)	190 (56 to 411)	2.39 (0.71 to 5.17)	-0.28 (-0.33 to -0.24)

AAPC, average annual percentage change; ASIR, age-standardized incidence rate; ASYR, age-standardized YLD rate; YLDs, years lived with disability.

Within parentheses were 95% uncertainty intervals for numbers, ASIRs, and ASYRs, and 95% confidence intervals for AAPCs, respectively.

Table S4. SDI-based epidemiology of eye injury among children and adolescents, 1990-2021.

location	Incidence					YLDs				
	**Number, 1990**	**ASIR per 100000, 1990**	**Number, 2021**	**ASIR per 100000, 2021**	**AAPC, 1990-2021**	**Number, 1990**	**ASYR per 100000, 1990**	**Number, 2021**	**ASYR per 100000, 2021**	**AAPC, 1990-2021**
High SDI	2408452 (1730868 to 3235320)	946.66 (681.89 to 1267.2)	1942465 (1345539 to 2692854)	823.34 (573.05 to 1136.02)	-0.44 (-0.5 to -0.39)	17696 (5242 to 37751)	6.96 (2.06 to 14.84)	14272 (4206 to 31202)	6.05 (1.79 to 13.2)	-0.44 (-0.5 to -0.39)
High-middle SDI	2636331 (1847441 to 3695227)	700.26 (492.52 to 978.12)	1686241 (1176401 to 2350032)	547.92 (383.66 to 761.25)	-0.78 (-0.9 to -0.65)	19348 (5712 to 41947)	5.14 (1.52 to 11.12)	12385 (3673 to 27358)	4.02 (1.2 to 8.87)	-0.78 (-0.9 to -0.65)
Middle SDI	3548474 (2397741 to 5176844)	460.09 (311.89 to 669.25)	2810594 (1864713 to 4159963)	368.57 (245.66 to 543.67)	-0.69 (-0.87 to -0.51)	26032 (7711 to 57804)	3.38 (1 to 7.49)	20636 (6066 to 46807)	2.71 (0.8 to 6.12)	-0.69 (-0.86 to -0.51)
Low-middle SDI	2600761 (1834422 to 3562909)	444.81 (312.66 to 611.38)	2756465 (1904317 to 3872390)	356.84 (247.11 to 499.85)	-0.77 (-0.97 to -0.57)	19086 (5809 to 41706)	3.26 (0.99 to 7.15)	20241 (6002 to 44284)	2.62 (0.78 to 5.72)	-0.76 (-0.96 to -0.57)
Low SDI	1314755 (960991 to 1752779)	477.68 (346.42 to 641.13)	2342172 (1671259 to 3177585)	402.95 (287.01 to 547.77)	-0.56 (-1.14 to 0.02)	9642 (2975 to 20523)	3.5 (1.08 to 7.48)	17183 (5293 to 36675)	2.96 (0.91 to 6.32)	-0.56 (-1.13 to 0.01)

AAPC, average annual percentage change; ASIR, age-standardized incidence rate; ASYR, age-standardized YLD rate; YLDs, years lived with disability.

Within parentheses were 95% uncertainty intervals for numbers, ASIRs, and ASYRs, and 95% confidence intervals for AAPCs, respectively.

Table S5. The ASIR and ASYR of eye injury among children and adolescents by region and cause in 2021.

Location	ASIR per 100000				ASYR per 100000			
	Unintentional injuries	Self-harm and interpersonal violence	Transport injuries	All injuries	Unintentional injuries	Self-harm and interpersonal violence	Transport injuries	All injuries
Global	407.26	22.14	4.84	434.24	2.99	0.16	0.04	3.19
Central Asia	556.29	10.50	4.57	571.36	4.09	0.08	0.03	4.20
East Asia	336.01	12.96	4.79	353.76	2.46	0.10	0.03	2.59
South Asia	322.23	12.20	2.48	336.91	2.37	0.09	0.02	2.48
Southeast Asia	198.79	19.05	3.80	221.64	1.46	0.14	0.03	1.63
High-income Asia Pacific	790.76	23.86	7.61	822.23	5.81	0.18	0.06	6.04
Central Europe	836.07	22.13	5.70	863.89	6.15	0.16	0.04	6.35
Eastern Europe	570.86	16.58	7.23	594.67	4.20	0.12	0.05	4.37
Western Europe	1099.48	20.85	8.41	1128.74	8.08	0.15	0.06	8.29
Central Sub-Saharan Africa	322.62	23.75	6.44	352.81	2.37	0.17	0.05	2.59
Eastern Sub-Saharan Africa	360.32	31.25	2.65	394.21	2.64	0.23	0.02	2.89
Southern Sub-Saharan Africa	347.53	19.33	4.61	371.47	2.55	0.14	0.03	2.73
Western Sub-Saharan Africa	369.27	21.73	3.21	394.21	2.71	0.16	0.02	2.89
North Africa and Middle East	350.48	75.20	9.05	434.73	2.58	0.55	0.07	3.19

High-income North America	687.04	16.20	13.93	717.16	5.05	0.12	0.10	5.27
Caribbean	573.34	17.33	5.59	596.26	4.21	0.13	0.04	4.38
Andean Latin America	502.39	4.32	4.34	511.05	3.69	0.03	0.03	3.75
Central Latin America	528.46	12.00	5.45	545.90	3.88	0.09	0.04	4.01
Southern Latin America	1251.64	18.24	9.73	1279.61	9.19	0.13	0.07	9.40
Tropical Latin America	552.41	17.42	6.04	575.87	4.05	0.13	0.04	4.22
Oceania	165.34	22.89	4.94	193.17	1.22	0.17	0.04	1.42
Australasia	1468.57	17.58	7.76	1493.91	10.78	0.13	0.06	10.97

ASIR, age-standardized incidence rate; ASYR, age-standardized YLD rate; YLD, year lived with disability.
